# Comparative transcriptome analysis of *Citrus macrophylla* tree infected with *Citrus tristeza virus* stem pitting mutants provides new insight into the role of phloem regeneration in stem pitting disease

**DOI:** 10.3389/fpls.2022.987831

**Published:** 2022-10-04

**Authors:** Maryam Khalilzadeh, Kyle Clark Weber, Manjul Dutt, Choaa Amine El-Mohtar, Amit Levy

**Affiliations:** ^1^Citrus Research and Education Center, University of Florida, Lake Alfred, FL, United States; ^2^Department of Plant Pathology, University of Florida, Gainesville, FL, United States

**Keywords:** *Citrus tristeza virus* (CTV), stem-pitting, secondary vascular tissue regeneration, transcriptome (RNA-seq), virus-plant interaction

## Abstract

Stem pitting is a complex and economically important virus-associated disease of perennial woody plants. Molecular mechanisms and pathways occurring during virus-plant interaction that result in this phenomenon are still obscure. Previous studies indicated that different *Citrus tristeza virus* (CTV) mutants induce defined stem pitting phenotypes ranging from mild (CTVΔp13) to severe (CTVΔp33) in *Citrus macrophylla* trees. In this study, we conducted comparative transcriptome analyses of *C. macrophylla* trees infected with CTV mutants (CTVΔp13 and CTVΔp33) and a full-length virus in comparison to healthy plants as control. The mild CTV stem pitting mutant had very few differentially expressed genes (DEGs) related to plant defense mechanism and plant growth and development. In contrast, substantial gene expression changes were observed in plants infected with the severe mutant and the full-length virus, indicating that both the p13 and p33 proteins of CTV acted as a regulator of symptom production by activating and modulating plant responses, respectively. The analysis of transcriptome data for CTVΔp33 and the full-length virus suggested that xylem specification has been blocked by detecting several genes encoding xylem, cell wall and lignin degradation, and cell wall loosening enzymes. Furthermore, stem pitting was accompanied by downregulation of transcription factors involved in regulation of xylem differentiation and downregulation of some genes involved in lignin biosynthesis, showing that the xylem differentiation and specification program has been shut off. Upregulation of genes encoding transcription factors associated with phloem and cambium development indicated the activation of this program in stem pitting disease. Furthermore, we detected the induction of several DEGs encoding proteins associated with cell cycle re-entry such as chromatin remodeling factors and cyclin, and histone modification. This kind of expression pattern of genes related to xylem differentiation and specification, phloem and cambium development, and cell cycle re-entry is demonstrated during secondary vascular tissue (SVT) regeneration. The microscopy analysis confirmed that the regeneration of new phloem is associated with stem pitting phenotypes. The findings of this study, thus, provide evidence for the association between stem pitting phenotypes and SVT regeneration, suggesting that the expression of these genes might play important roles in development of stem pitting symptoms. Overall, our findings suggest that phloem regeneration contributes to development of stem pitting symptoms.

## Introduction

Stem pitting (SP) is a common virus-induced phenotype that has been reported in perennial woody plants, including numerous stone fruits, apples, grapevines, avocado, and citrus (Menge and Ploetz, 2003; Amenduni et al., 2005; Moreno et al., 2008; Meng and Rowhani, 2017; Komorowska et al., 2019). This phenotype results from interference with stem growth. The stem-pitted area is a mosaic of altered and normal tissues. In normal tissues of infected trees, the cambium divides and differentiates normally and produces new xylem on the inward side and new phloem on the bark side, while in altered tissues the development of xylem and phloem is disrupted. The surrounding areas grow normally, leaving the disrupted areas as indented areas or pits (Dawson et al., 2015). Different viruses from the plant virus taxon induce SP in a range of perennial woody plants. In citrus, a member of the family *Closteroviridae* causes SP disease that greatly limits the production of susceptible citrus varieties to this malady in many citrus industries around the world. SP disease leads to lack of vigor and growth reduction, and yield unmarketable fruits that cause high cumulative economic losses.

*Citrus tristeza virus* (CTV), a member of the family *Closteroviridae*, is the causal agent responsible for several citrus diseases characterized by mild to severe symptoms. CTV has long flexuous particles 2,000 nm long and 12 nm wide, and has a single-stranded, positive-sense genomic RNA (gRNA) of 19.3 kb. CTV causes three types of syndromes, namely, yellow seedlings, SP, and quick decline in citrus based on virus strains, host cultivars, and environmental conditions (Bar-Joseph et al., 1989). CTV SP, which results from interference in stem growth, takes place in most citrus hosts under proper conditions and can develop in many types of scions regardless of rootstocks (Dawson et al., 2013). Symptoms of longitudinal pits can be found in the wood under the bark of the main trunk, branches, and twigs. Infected trees are stunted and bear fewer fruits that are poor in quality and size. The SP syndrome is a complex disease with a high degree of specificity between virus isolates and different citrus species and varieties, such that some CTV isolates cause SP in some citrus species but not in others (Moreno et al., 2008; Dawson et al., 2015). Additionally, there is a continuum of different phenotypes of SP from large stem pits that are visible in tree trunks to a high density of very small pits (Garnsey et al., 2005; Hilf et al., 2005).

VT, T3, and T68 are isolates associated with SP induced by CTV (Harper and Cowell, 2016; Selvaraj et al., 2019). Brlansky et al. (2002) examined the cytological changes in stem tissues of *sweet orange* caused by a severe CTV-SP isolate by light and electron microscopy. He demonstrated that there is lack of normal cambium in pitted areas that results in lack of production of normal xylem and overproduction of phloem and phloem parenchyma tissue in this area (Brlansky et al., 2002). Tatineni and Dawson (2012) studied the effect of deletion of three non-conserved p13, p18, and p33 CTV genes on symptom phenotypes. They showed that the full-length virus causes stem pitting symptoms in *Citrus macrophylla* (susceptible experimental host), that deletion of p33 and p18 increased stem pitting symptoms, and that deletion of p13 reduced stem pitting. Therefore, the stem-pitting phenotype results from a balance between the expressions of different viral genes. In areas of stem pits, the virus induces changes in cellular differentiation and development (Tatineni and Dawson, 2012). Furthermore, it was shown that in this area, CTV infects unspecialized xylem cells (Sun and Folimonova, 2019). In a recent study, the development of stem pitting in *C. macrophylla* infected with a CTV wild type and the p33 deletion mutant was studied by time-course observations and histological analyses. The development of stem pitting was shown to be associated with the viral ability to infect xylem and prevents them from maturing by interrupting cellular lignification (Sun and Folimonova, 2019).

The SP syndrome generally does not cause tree death but reduction in yield, which causes economic losses in an increasing number of countries (Moreno et al., 2008). Despite the prevalence of SP in a range of plant species and specifically the economic importance of this syndrome to the citrus industry, the underlying molecular mechanisms and pathways occurring during virus-plant interactions that result in stem pitting symptom development are still unclear.

Previously, Tatineni and Dawson (2012) reported that different CTV mutants induce defined stem pitting phenotypes in *C. macrophylla*. To understand the molecular mechanism behind this phenomenon, we took advantage of CTV mutant lines (Tatineni and Dawson, 2012) to compare the gene expression profile of the defined stem pitting phenotypes. We conducted a transcriptome profiling analysis on infected trees with CTVΔp13 that induces no visible stem pits or milder symptoms than the full length, CTVΔp33 that increases the stem pitting phenotype, and full-length CTV. We highlighted the p13 and p33 proteins of CTV as regulators of CTV stem pitting phenotype and symptom development in the later stage of infection by activating and modulating host responses. We demonstrate that genes related to secondary vascular tissue (SVT) regeneration are differentially expressed in the CTV full-length and mutant line with severe stem-pitting phenotype, suggesting the involvement of SVT regeneration in stem pitting symptom development. Furthermore, the microscopy analysis confirmed the association between stem pitting symptom development and phloem regeneration.

## Materials and methods

### Plant materials and inoculation of citrus plants

The full-length cloned CTV, CTVΔp13, and CTVΔp33 were previously produced from cDNA constructs (Tatineni et al., 2008). The CTV variants were maintained in *C. macrophylla* in a greenhouse (Tatineni and Dawson, 2012) located at Citrus Research and Education Center, University of Florida (Lake Alfred, FL, United States). A minimum of three healthy *C. macrophylla* plants (2 years old) were graft-inoculated with buds from infected trees with CTV variants as biological replicates for each treatment. Buds from healthy plants were used to mock-inoculate the healthy controls. All the plants were maintained in a temperature-controlled greenhouse. CTV infection of test plants was determined at 3 months post inoculation (mpi) by analyzing extracts from young leaf midribs by RT-PCR and enzyme-linked immunosorbent assay (ELISA). Bark sampling of healthy and infected plants at 8 months post-inoculation (mpi) was performed.

### Extraction of total RNA

Bark tissue samples (100 mg) from the main stem of three independent replicates of CTV- infected and healthy plants were collected, and the total RNA of each sample was extracted using the TRIzole reagent (Invitrogen, CA, United States) following the manufacturer's protocol. RNA samples were quantified and qualified using the NanoDrop™ 1000 Spectrophotometer (Thermo Fisher Scientific Inc., Waltham, MA, United States) and then treated with DNase to remove the residual genomic DNA. The integrity of the RNA was examined using the Agilent 2100 Bioanalyzer (Agilent Technologies Inc., Waldbronn, Germany).

### Library construction and RNA sequencing

One microgram of high-quality total RNA with an RNA integrity number (RIN) value above 5 was used for library preparation and RNA sequencing using the TruSeq Stranded Total RNA Library Prep Kit (Illumina) and following the manufacturer's instructions. The cDNA libraries were then subjected to generate paired-end reads using an Illumina NovaSeq6000 sequencing platform. In this experiment, total reads per library yielded ranged from 41 to 62 million reads with a mean number of 48 million reads per sample. Library construction and RNA sequencing were performed in the ICBR NextGen DNA Sequencing and Gene Expression cores facility at the University of Florida. The RNA-seq raw data of all the samples were deposited in NCBI BioProject PRJNA851012 with accession numbers of 4 objects of SRR19781992, SRR19781993, SRR19781994, and SRR19781995 that correspond to CTVΔp33, full-length CTV, CTVΔp13, and *C. macrophylla* healthy trees, respectively.

### Differential gene expression analysis

Reads acquired from the Illumina NovaSeq 6000 platform were cleaned up with the cutadapt program (Martin, 2011) to trim off sequencing adaptors and low-quality bases with a quality Phred-like score < 20. Reads < 40 bases were excluded from the RNA-seq analysis. The genome of *Citrus sinenesis* from the citrus genome database (https://www.citrusgenomedb.org/citrus_downloads/Citrus_sinensis/C.sinensis_Hzau_v2.0_genome/) was used as the reference sequence for the RNA-seq analysis. The cleaned reads of each sample were mapped to the reference sequences using the read mapper of the STAR package (Spliced Transcripts Alignment to a Reference, v2.7.9a) (Dobin et al., 2013). The mapping results were processed by HTSeq (high-throughput sequence) analysis in Python, v0.11.2 (Anders et al., 2015), computational applications SAMtools, and scripts developed in-house at ICBR of UF to remove potential PCR duplicates and to choose and count uniquely mapped reads for gene expression analysis. The counted reads of each gene were analyzed with a DESeq2-based R pipeline. Significant up- and downregulated genes were selected based on their Log2 fold change and *P* adjusted value for downstream analysis. A minimal cutoff of |Log2fold| ≥ 2 and *P*-adj < 0.05 was applied to ensure that significant DEGs were selected, and all replicates of a sample had to meet this cutoff. Expressed genes in each sample were then compared utilizing a Bioinformatics and Evolutionary Genomics Venn diagram maker (http://bioinformatics.psb.ugentbe/webtools/Venn/).

### Annotation analysis

The statistically significant DEGs were further analyzed for functional annotation. A Gene Ontology (GO) enrichment analysis was performed using agriGO v2 (Tian et al., 2017) to annotate the DEGs at three levels of biological process, molecular function, and cellular component. The statistical test Fisher, the multi test adjustment Yekutieli, a significance level of 0.05, and a minimum mapping of 5 were selected as parameters. The significantly assigned GO terms from agriGO were then inserted into the REVIGO software to remove redundant GO terms (Supek et al., 2011).

### MapMan analysis

To visually understand the functional and pathway differences between samples and to find the role of specific gene in a metabolic pathway, A MapMan analysis (Thimm et al., 2004) was performed using a MapMan program, version 3.6.0RC1. A custom MapMan mapping file was generated from a BLASTP bi-directional best hit comparing *Arabidopsis thaliana* (https://www.arabidopsis.org/download_files/Sequences/TAIR10_blastsets/TAIR10_pep_20101214) and *Sweet orange* (https://www.citrusgenomedb.org/citrus_downloads/Citrus_sinensis/C.sinensis_Hzau_v2.0_genome/) (Camacho et al., 2009; Wang et al., 2014). The BLASTP thresholds utilized in determining similarity between genes had a sequence coverage >0.7, identity > 30%, e value < 1e-10, and bit-score > 60. The statistically significant DEGs for each treatment and their Log2 fold changes were placed into an xls file and imported into the MapMan program, selecting the BLASTP-generated mapping file as the mapping. The DEGs were classified into MapMan BINs, and their annotated functions were visualized by mapping the DEGs against the custom mapping file.

### RNA-seq analysis validation by RT-qPCR

To validate the results of DEGs identified by RNA-seq, the expression level of genes involved in lignin monomer synthesis like cinnamoyl-CoA reductase (CCR), caffeic acid O-methyltransferase (COMT), and 4-coumarate: CoA ligase (4CL) were estimated by reverse transcription quantitative polymerase chain reaction (RT-qPCR) in CTV mutant-lines infected and healthy control trees. The citrus ubiquitin 10 (UBQ 10) gene was used as the reference gene, and three replicates for each reaction were prepared. Gene-specific primers and methods were previously designed and described (Sun and Folimonova, 2022).

### Microscopy analysis

For microscopy analysis, young stems from three biological replicates of infected plants with different CTV mutants and healthy plants were sampled. Then, they were dissected into very thin sections with single edged disposable blades and were placed on a drop of water on a microscope slide with a coverslip. To estimate the thickness of the developing phloem, the vasculature tissues in each sample were analyzed by light microscopy, and phloem width was quantitatively measured. The mean values of phloem width measurements from healthy and infected plants were calculated using R and compared using an ANOVA function.

## Results

### Increased stem pitting symptoms were associated with differentially expressed genes enrichment

**Figure 1:**
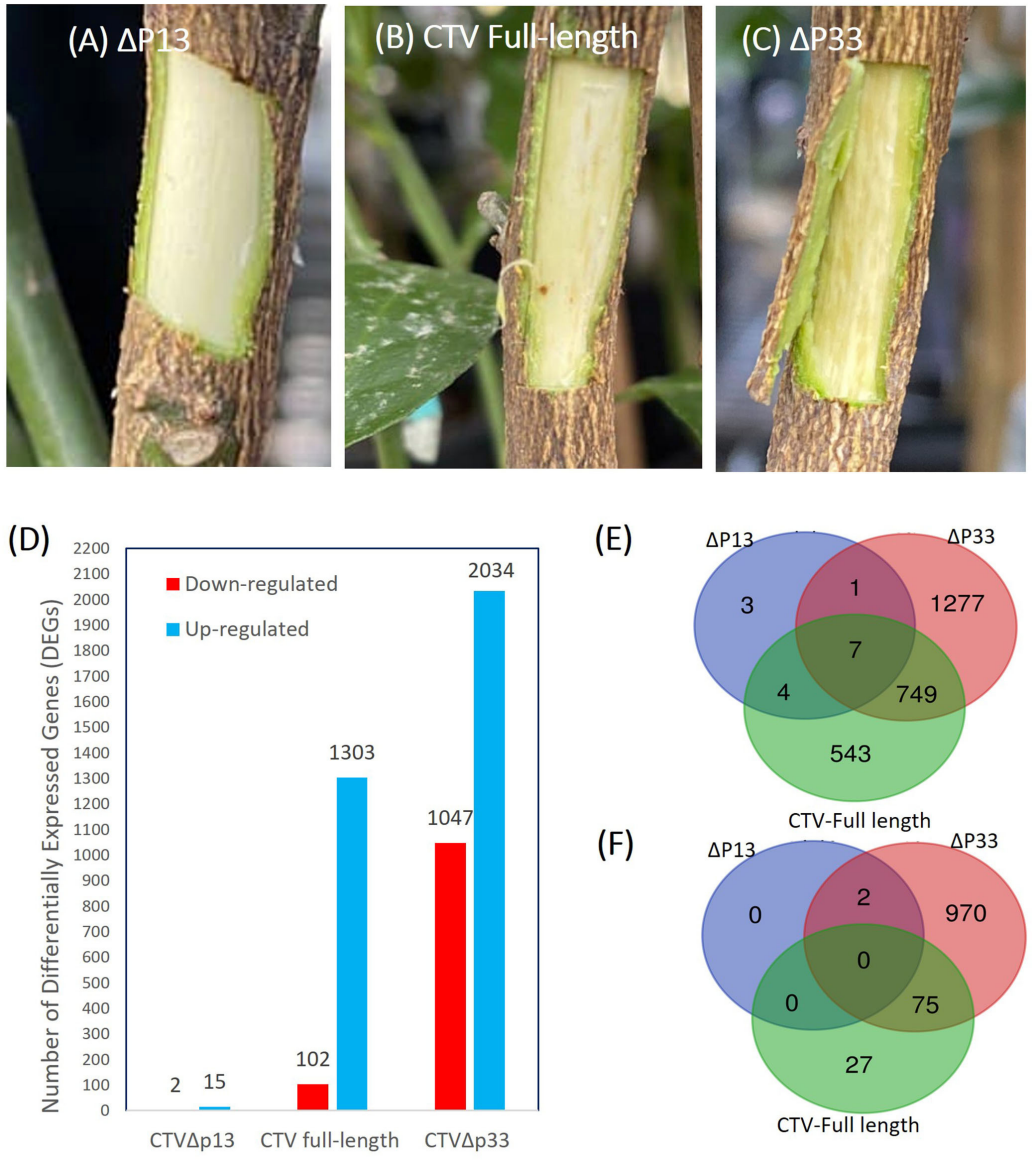
Increase in the stem pitting symptoms of the CTV-infected *C. macrophylla* trees is associated with increase in the number of up- and downregulated genes. **(A–C)** Stem pitting symptoms in the experimental host at 8 months post-inoculation. **(A)** CTVΔp13 induced no visible stem pits. **(B)** The full-length virus-induced moderate stem pits. **(C)** CTVΔp33 induced severe stem pits in the main stem. **(D)** Number of up- and downregulated genes for each pairwise comparisons. Venn diagrams demonstrate numbers of unique and common **(E)** upregulated and **(F)** downregulated differentially expressed genes (DEGs) between *Citrus macrophylla* trees infected with CTVΔp13, full-length CTV, and Δp33 vs. not infected trees.

We infected the experimental host, *C. macrophylla*, with full-length CTV, CTVΔp13, and CTVΔp33. The CTV infection of the trees was confirmed at 3 mpi by ELISA and RT-PCR. We looked for symptom development at 8 mpi. The isolate of CTVΔp13 caused no visible stem pitting symptoms in the experimental host, while the full-length virus caused sporadic pits, and deletion of p33 induced severe stem pitting symptoms with a considerable increase in the number of pits compared to the full-length (Figure 1). The qPCR confirmed there was no difference in the amount of the virus (Supplementary Figure 1). We grouped the CTV mutants into three distinct phenotypes of mild (CTVΔp13), moderate (full length), and severe (CTVΔp33) based on symptoms. The phenotypes were consistent with the observations made by Tatineni and Dawson (2012). For the transcriptome profiling analysis, three pairwise comparisons, namely, CTVΔp13 vs. healthy control (Δp13 vs. H), full-length CTV vs. healthy control (full vs. H), and CTVΔp33 vs. healthy control (Δp33 vs. H) were performed using criteria log_2_ fold change in the range ≥ +2 and ≤ −2 and corrected *P* < 0.05.

The transcriptome analysis of the different stem pitting phenotypes of CTV-infected trees identified a total 4,503 DEGs in all the groups. A total of 520 of the differentially regulated genes did not show a similarity with other sequences in databases, and they might be genes-specific for *C. macrophylla* or non-protein coding sequences. Considerable changes in the number of differentially expressed genes were detected across two sample sets of full-length CTV and the severe stem-pitting phenotype. At 8 months post-inoculation, there were more DEGs in response to infection with CTVΔp33 than to the CTV full-length. The large numbers of up- and downregulated transcripts from this sample set showed extensive changes in transcriptome during the development of stem pitting. A total of 1,405 DEGs in full-length CTV and 3,081 in Δp33 were detected (Figure 1). A dramatically lower number of DEGs were detected from CTVΔp13. The number of DEGs for the mutant with mild symptom were considerably lower than for CTV full-length or the mutant with severe phenotype, with a total of 17 DEGs in Δp13 (Figure 1). The Venn diagram demonstrates the number of up- and down-regulated genes in each individual sample set and the number of common DEGs among all the groups (Figure 1).

**Figure 2:**
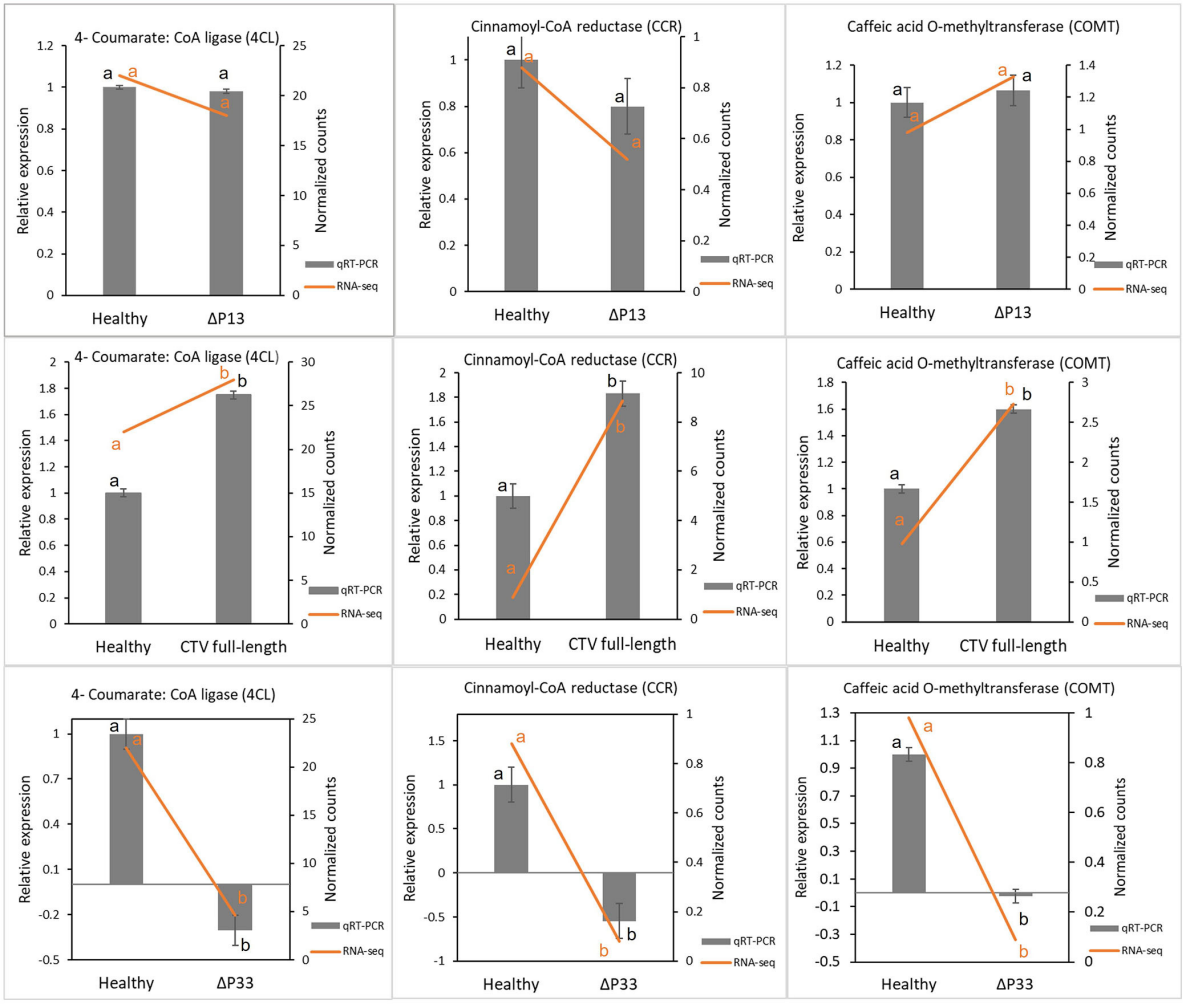
Validation of transcriptome results by reverse transcription quantitative PCR (RT-qPCR). Some genes involved in lignin monomer synthesis were selected for RT-qPCR verification. Expression levels of tested genes were normalized based on transcript levels of ubiquitin 10 (UBQ 10) gene. Different letters (a and b) represent a significant difference at *p* ≤ 0.05. Error bars represent standard deviations of the means (*n* = 3).

To validate the RNA-seq results, genes involved in lignin monomer synthesis were selected for RT-qPCR verification. These genes were selected as controls, since their expression was already shown to decrease with CTVΔp33 infection (Sun and Folimonova, 2019). The citrus UBQ 10 gene was used as the reference gene. The expression levels of selected genes were calculated for all the treatments using the 2^−Δ*Δct*^ method. The results of the RT-qPCR analyses indicated that expression trends were similar to those from RNA-seq, and that they were consistent with the transcriptome data (Figure 2).

### Mild SP mutant induced few general defense and development response DEGs

**Figure 3:**
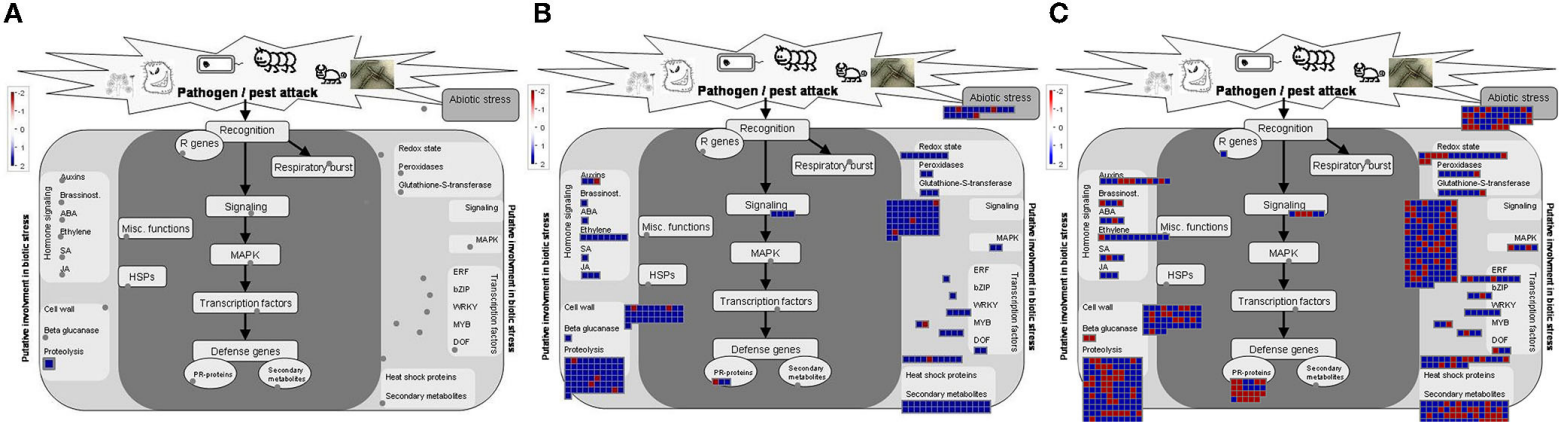
MapMan visualization of differential expressed genes related to biotic stress overview, **(A)** CTVΔp13 vs Healthy, **(B)** full-length CTV vs Healthy, and **(C)** CTVΔp33 vs Healthy. Blue and Red squares indicate up- and down regulation of genes respectively.

A Gene Ontology (GO) analysis was performed to identify functional categories of DEGs. For the mild stem pitting mutant, no GO terms were obtained. A MapMan analysis was performed to get further insights into the DEGs. MapMan diagrams for this mutant indicated few DEGs related to biotic stress, proteasome and autophagy, protein synthesis, metabolism, and transcription factors. In the biotic stress diagram for the mild stem pitting phenotype, only one up-regulated gene (Cs7g31440) related to proteolysis was identified (Figure 3). In the cell function overview, there were few DEGs related to development (cs1g09660), protein and amino-acid synthesis (cs9g07210), protein modification, and regulation of transcription (cs3g19950 and cs7g06730) for Δp13 (Supplementary Figure 2). The mild mutant showed few DEGs common with the other two phenotypes including NAC domain-containing protein transcription factor, alternative oxidase, RNA-dependent RNA polymerase 1, serine/threonine protein kinase family protein, and 60s ribosomal protein L19, which have a role in development, defense response, and protein synthesis, respectively (Table 1).

**Table 1:** Differentially expressed genes (DEGs) for *Citrus macrophylla* trees infected with CTVΔp13 (mild stem pitting mutant).

	**Identifier**	**Description**
**Down-regulated genes**	Cs9g02070	Zinc finger protein-related
	Cs7g06730	Chloroplast nucleoid DNA-binding protein
**Up-regulated genes**	Cs1g09660	NAC domain-containing protein Transcription factor
	Cs7g08680	Unknown protein
	Cs9g07210	60S ribosomal protein L19 (RPL19B)
	Cs3g18740	F-box family protein
	Cs5g12960	Serine/threonine protein kinase family protein
	orange1.1t04732	Putative RNA-dependent RNA-polymerase
	Cs3g09710	Alternative oxidase 1D
	Cs2g11100	Unknown protein
	orange1.1t01039	Transcriptional regulator
	Cs5g23340	Putative S1 RNA binding domain protein
	Cs3g19950	Transcription regulatory protein SWI3
	Cs2g22900	NADH dehydrogenase/ disulfide oxidoreductase
	Cs4g12210	Not assigned. unknown
	Cs7g31440	Translation initiation factor
	orange1.1t02600	WRKY transcription factor

Gene identifier, functional grouping, and gene description are based on the gene ontology in the MapMan program (Thimm et al., 2004).

### Extensive DEGs from different pathways were identified in moderate and severe phenotypes

**Figure 4:**
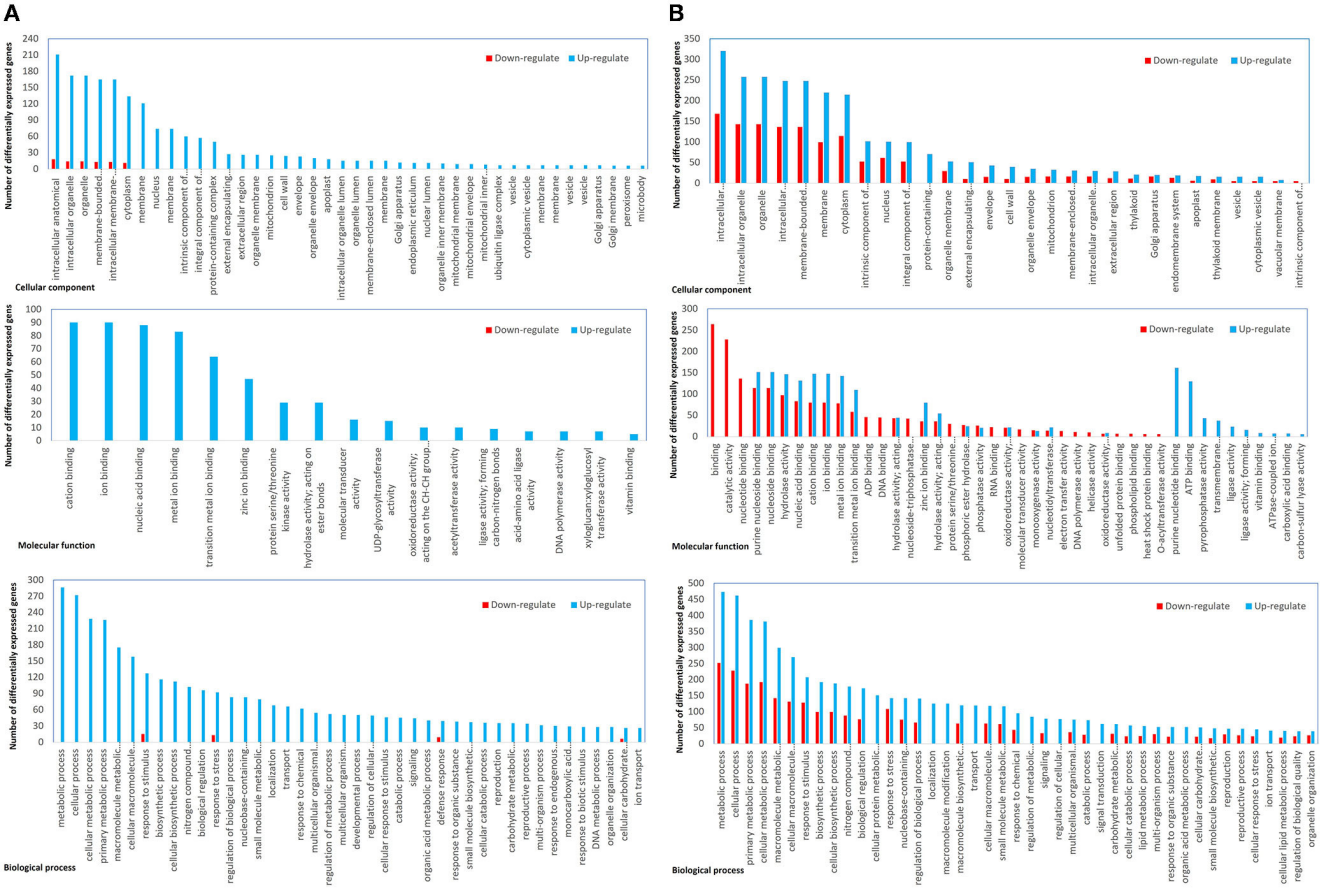
Functional annotation of differential expressed genes. **(A)** Full-length CTV infected vs not infected and **(B)** CTVΔp33 infected vs not infected *Citrus macrophylla* trees. The genes were divided into three categories: Cellular component, molecular function, and biological process genes.

In the sample sets of full-length CTV and the severe mutant, all DEGs were assigned into three categories, namely, biological process (152), molecular function (47), and cellular component (40) (Figure 4). The MapMan diagrams indicated that for full-length CTV and the severe stem-pitting phenotype, biotic stress, metabolism, large enzyme families, transcription factors, proteasome and autophagy, protein targeting, as well as receptor-like kinases, were all enriched (Supplementary Figures 3–11). In the biotic stress overview, secondary metabolites, cell wall biosynthesis, signaling, proteolysis, abiotic stress, transcription factors, and redox state-related were enriched. Among them, proteolysis, signaling, secondary metabolism, and cell wall and abiotic stress-associated genes were the most enriched in the CTV- infected trees with full-length virus and severe stem-pitting phenotype (Figure 3).

The transcriptome data of full-length CTV and CTVΔp33 indicated several DEGs encoding proteins involved in plant-pathogen interaction. In pathogen-associated molecular pattern (PAMP)-triggered immunity, one glycerol kinase (NHO) inducing defense related gene and calcium signaling DEGs including calmodulin binding protein, calcium binding protein (CaMCML), calcium-dependent protein kinases (CDPKs), and cyclic nucleotide-gated ion channel (CNGC) proteins were identified (Table 2). The overexpression of genes encoding the LRR receptor-like protein kinase FLS2 was indicated in full-length CTV. Additionally, several DEGs involved in effector-triggered immunity (ETI) including RPM1-interacting protein 4 (RIN4), disease resistance protein RPM1, probable serine/threonine protein kinase PBL7, and HSP90 were reported in the severe stem pitting phenotype (Supplementary Table 1). In CTVΔp33, 28 DEGs related to leucine-rich repeats receptor-like kinases (LRRs-RLKs) were identified, and 18 out of them were upregulated, whereas in full-length CTV, 12 LRRs-RLKs genes were up- and only one were downregulated (Supplementary Table 1). LRRs had been reported to have a role in stress response and plant-microbe interactions (Shiu and Bleecker, 2001). The expression of several genes coding for pathogenesis-related proteins (PRs) including TIR-NBB-LRR class, NBS-LRR class, and CC-NBS-LRR class genes was modulated in response to CTV-full and CTVΔp33 infection (Supplementary Table 1). Plant infection with these stem-pitting phenotypes induced heat-shock proteins (HSPs) and genes encoding proteins with oxidant and antioxidant activity. Additionally, few downregulated genes related to reactive oxygen specious (ROS) production and ROS detoxification were detected (Supplementary Table 1).

**Table 2:** Differentially expressed genes (DEGs) encoding proteins involved in pathogen-associated molecular pattern (PAMP)- triggered immunity for *Citrus macrophylla* trees infected with full-length CTV and CTVΔp33 (moderate and severe stem pitting phenotypes).

	**Identifier**	**log_2_Fold change**	**Description**
**CTV full-length**	orange1.1t01393	2.740019	Glycerol kinase
	Cs9g18200	4.246887	Cyclic nucleotide-gated ion channel
	Cs2g16550	4.570408	Calcium-dependent phosphotriesterase-like protein
	Cs9g05900	3.810352	Calcium binding motif-containing protein
	cs4g10400	2.651	Calcium ion binding / calmodulin-dependent protein kinase
	cs7g27130	2.626	Calmodulin binding
	cs4g01630	2.747	Calmodulin-binding protein
	cs2g05140	3.646	Calmodulin binding
	cs7g06350	4.927	Calmodulin-binding heat-shock protein-related
	cs7g07560	−2.161	Calcium-binding protein
	cs5g16170	2.676	Calcium-binding EF hand family protein
	cs4g03040	6.824	Calmodulin-binding protein
**CTV** **ΔP33**	orange1.1t01393	4.080133	Glycerol kinase
	Cs9g18200	7.111967	Cyclic nucleotide-gated ion channel
	Cs7g07060	−3.20757	Probable cyclic nucleotide-gated ion channel
	Cs9g18260	−2.76939	Cyclic nucleotide-gated ion channel
	cs4g10400	3.644	Calcium ion binding / calmodulin-dependent protein kinase
	cs4g10430	3.095	Calcium ion binding / calmodulin-dependent protein kinase
	cs7g27130	2.861	Calmodulin binding
	cs3g11350	2.7	Calcium-binding EF hand family protein
	cs9g02980	−2.715	Calmodulin-binding family protein
	cs4g10490	3.856	Calmodulin-dependent protein kinase
	cs2g26210	−2.768	Calcium-transporting ATPase/ calmodulin binding
	cs6g13010	−2.036	Calmodulin-binding family protein
	cs7g06350	2.527	Calmodulin-binding heat-shock protein-related
	cs9g05820	7.752	Calcium-binding EF hand family protein
	cs9g05900	4.042	Calcium-binding EF hand family protein
	cs6g10520	2.693	Calcium-binding protein
	cs7g07560	−2.052	Calcium-binding protein
	cs2g07910	2.749	Binding / calmodulin binding
	cs4g11690	−2.212	Calcium-binding EF hand family protein
	cs1g18400	−2.368	Calcium ion binding
	cs1g18050	5.396	Calmodulin binding
	cs6g21810	−3.239	Calmodulin-binding protein-related
	cs5g16170	3.431	Calcium-binding EF hand family protein
	cs4g03040	5.755	Calmodulin-binding protein
	cs9g16960	2.166	Calmodulin binding

Gene identifier, functional grouping, and gene description are based on the gene ontology in the MapMan program (Thimm et al., 2004).

Several genes encoding secondary metabolites such as alkaloid-like, simple phenols, betains, terpenoids, dihydroflavonols, flavonoids, tocopherol, flavonols, lignin and lignans, MVA pathway, non-MVA pathway, and phenylpropanoids were enriched in full-length CTV and Δp33. Phenylpropanoids, lignin and lignans, and terpenoids were the most enriched secondary metabolites. In full-length CTV-infected, DEGs related to secondary metabolism were upregulated, while for CTVΔp33 infection, both up- and downregulated genes were identified (Supplementary Table 1).

In total, 60 DEGs related to phytohormone synthesis and signal transduction pathways including jasmonic acid (JA), ethylene (ET), salicylic acid (SA), brassinosteroid (BR), abscisic acid (ABA), gibberellic acid (GA), auxin, and cytokinins were identified in two stem-pitting phenotypes (Supplementary Table 1). Among them, ET was the most enriched hormone. Forty-two out of the 60 DEGs were detected in CTVΔp33 with 32 up- and 10 downregulated genes. In contrast, there were 17 up- and one downregulated genes encoding hormones in full-length CTV. The gene expression profile of CTVΔp33- and full-length CTV-infected trees identified DEGs coding the auxin-responsive protein involved in auxin signal transduction. Furthermore, several genes related to abscisic acid and jasmonic acid signal transduction and salicylic acid and ethylene synthesis with a role in stress response were enriched in the stem-pitting phenotypes.

### DEGs related to secondary phloem regeneration contribute to stem pitting symptom development

**Figure 5:**
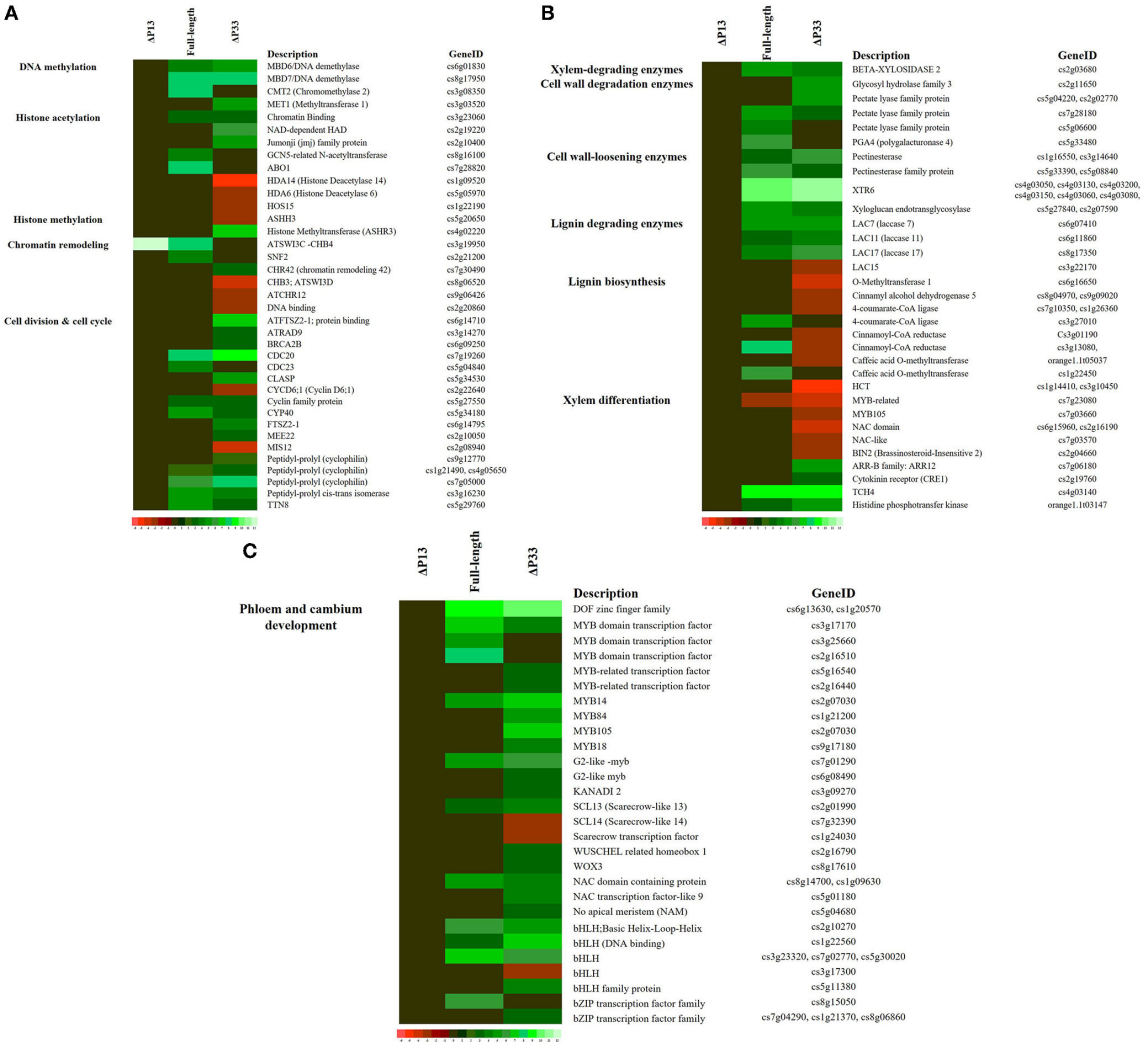
Expression pattern of some genes associated with **(A)** Cell cycle re-entry, **(B)** xylem specification, and **(C)** phloem and cambium development in *Citrus macrophylla* infected with CTV mutants during stem-pitting symptom development. Complete description for each gene can be found in Supplementary Table 1. Gene identifier, functional grouping and gene description is based on the gene ontology in the MapMan program (Thimm et al., 2004).

Our data showed the upregulation of genes related to regulation of DNA methylation in moderate and severe stem-pitting phenotypes. DEGs associated with histone acetyltransferase were identified in full-length CTV. Meanwhile, several genes involved in histone modification were differentially expressed in CTVΔp33 including histone acetylation and histone methylation-related genes. Interestingly, we identified upregulated genes associated with chromatin remodeling factors in full-length CTV. Simultaneously, several DEGs related to this factor were identified in Δp33. The expression of genes related to cell cycle and cell division such as cyclin family proteins was modulated in two CTV stem pitting phenotypes (Figure 5 and Supplementary Table 1).

In this study, a different expression pattern of some genes associated with xylem specification was observed. Upregulation of xylem-degrading, cell wall degradation, cell wall loosening, and lignin-degrading enzymes was observed in two CTV stem pitting phenotypes. Furthermore, downregulation of some genes involved in lignin biosynthesis such as 4-coumaroyl-CoA synthase, 4-coumarate-CoA ligase (4CL), hydroxycinnamoyl-CoA shikimate (HCT), cytochrome P450 (CYP98A3), caffeic acid O methyltransferase (COMT), cinnamoyl-CoA reductase (CCR), and cinnamyl-alcohol dehydrogenases (ATCAD4 and ATCAD5) were detected in Δp33 with the severe stem pitting phenotype. Our data demonstrated the downregulation of Brassinosteroid insensitive 2 (BIN2) in the severe stem pitting phenotype. Additionally, upregulation of the gene encoding xyloglucan-xyloglucosyl transferase (TCH4) related to brassinosteroid signal transduction was identified in full-length CTV and Δp33. There were upregulated genes associated with the cytokinin signal transduction pathway including cytokinin receptor (CRE1), histidine-containing phosphotransfer protein (AHP), and two component response regulators (ARR-B family) in CTVΔp33. In addition, AHP was upregulated in full-length CTV (Figure 5 and Supplementary Table 1). The transcriptomic data indicated enrichment of KANADI 2 (KAN2), G2-like myb family transcription factor, no apical meristem (NAM) family protein, DOF zinc finger family, MYB-related transcription factor family, Scarecrow transcription factor, WUSCHEL related homeobox, NAC domain containing protein, basic helix-loop-helix and bZIP transcription factor family during full-length CTV and CTVΔp33 infection (Figure 5 and Supplementary Table 1).

### Stem pitting symptom production is associated with phloem regeneration

**Figure 6:**
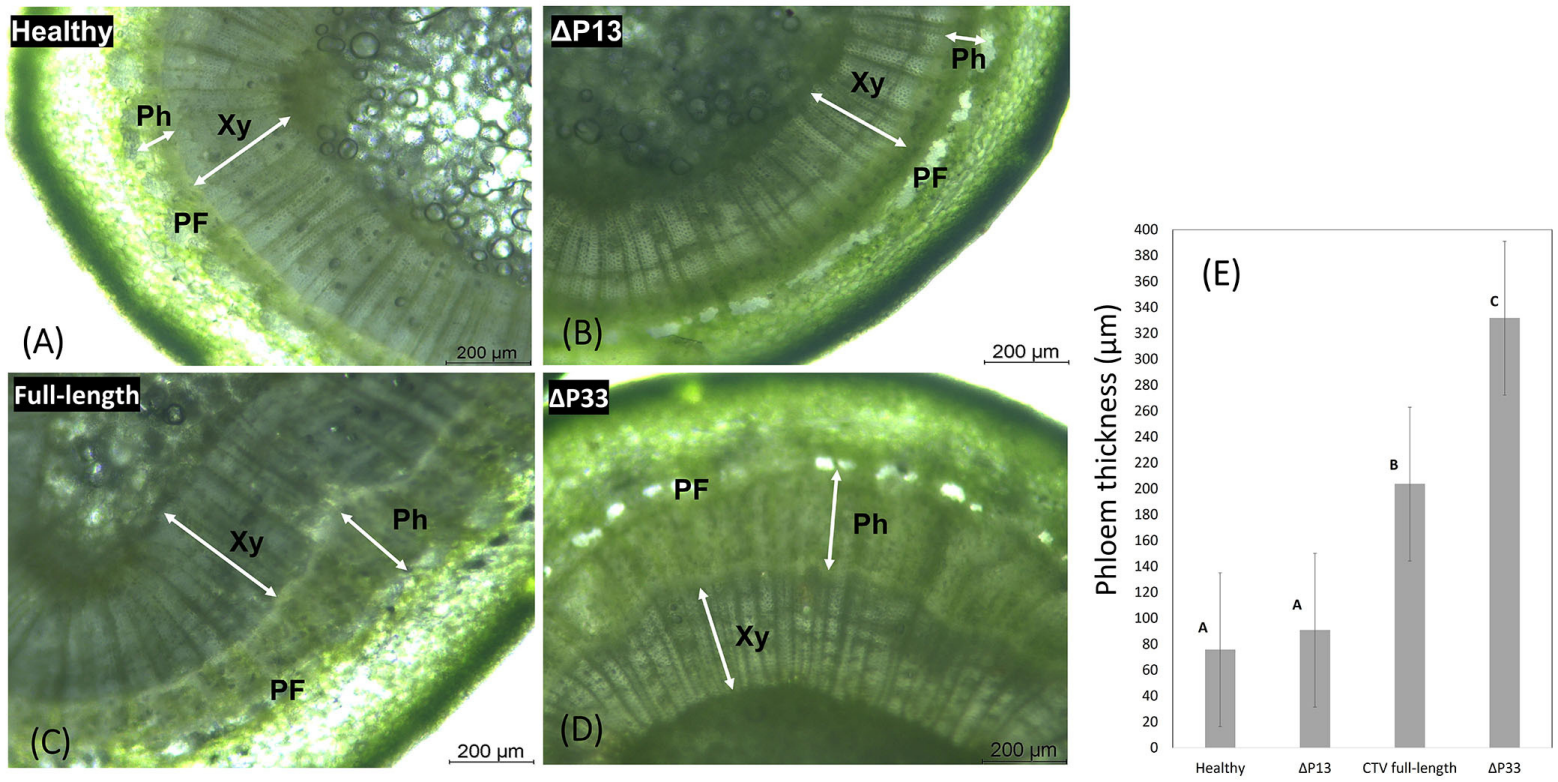
Transverse thin hand-cut sections of young stems from the healthy and infected *Citrus macrophylla* trees with CTV mutants showing the association of stem-pitting symptom development with secondary phloem regeneration. The photographs were taken under light microscope. **(A)** Healthy, **(B)** infected trees with CTVΔp13, **(C)** full-length CTV, and **(D)** CTVΔp33 respectively (40 × objective lens). Abbreviation: Xy, xylem; Ph, phloem; PF, phloem fiber. Scale bar = 200 μm. **(E)** Phloem width measurements of young stems from the healthy and infected *Citrus macrophylla* trees with CTV mutants. Shaded bars represent the mean values of phloem thickness (*n* = 20). The different letters denote statistically differences as determined by analysis of variance of mean values (*P* = < 0.05).

Transverse thin hand-cut sections of young stems from new branches were examined by light microscopy to measure the width of phloem tissues and make a comparison in secondary phloem tissue among plants infected with different CTV stem pitting phenotypes and non-infected plants. There was a thin layer of phloem tissue in the healthy and infected plants with mild stem pitting phenotype (Figure 6). In contrast, this tissue was thick in the moderate and severe phenotypes, and in areas that correspond to the pits, it was even wider than other places (Figure 6). The width of phloem tissues in CTV-infected plants with the full-length virus and severe phenotype was higher than that of the mild phenotype and healthy plants, and the difference was significant (Figure 6).

## Discussion

Previously, the effect of deletion of the p33 and p13 genes on stem pitting symptoms in *C. macrophylla* was reported in which CTVΔp33 causes more severe stem pitting symptoms than full-length CTV, while CTVΔp13 induces no visible or even milder symptoms than the full-length virus (Tatineni and Dawson, 2012). In this study, infected plants with Δp13, full-length CTV, and Δp33 induced different phenotypes. Among them, no visible stem pitting phenotype was seen in Δp13, while Δp33 showed severe degrees of stem pitting symptoms. All of the three subsets showed viral infection and replication in *C. macrophylla* cells. To understand the molecular mechanisms and putative metabolic pathways involved in symptom development and in decreasing/increasing stem pitting phenotype, we performed a comparative transcriptome analysis of *C. macrophylla* trees infected with the CTV mutants.

Substantial changes in the number of differentially expressed genes were observed in plants infected with CTVΔp33 and the full-length virus compared to the healthy trees. The stem-pitted area is a mosaic of altered and normal tissues, and we examined the mosaic tissue. So, the effect on the altered areas could be even greater than what we reported in this study. In contrast, considerably lower changes in gene expression were observed in plants infected with Δp13 that showed no SP symptoms. The gene annotation analysis of our study identified 239 GO categories, including 152 biological processes, 47 molecular functions, and 40 cellular components. In plants infected with full-length CTV, the majority of differential expressed genes were upregulated genes. Meanwhile, the downregulated genes in this group were annotated in only two GO categories of biological process and cellular components. On the other hand, the gene ontology analysis of CTVΔp33 demonstrated that all the three categories have both up- and downregulated genes, and the biological process category showed the highest number of DEGs. The RNA-seq data for CTVΔp13, which is considered to be a mild phenotype, showed few DEGs related to plant defense mechanism such as genes associated with ROS production and detoxification, protein degradation, WRKY transcription factor, and RNA-dependent RNA polymerase (RDR) 1. In addition, upregulation of few genes related to plant growth and development, and protein synthesis was detected. This result may suggest that the mild stem pitting phenotype induces few general defense response and physiology changes in plants.

Most of the DEGs observed in this study were related to the full-length CTV and severe stem pitting phenotypes. The changes were widespread across varied families and functions, hinting the complexity of the stem pitting phenotype. Majority of the DEGs were associated with biosynthesis of metabolites, biotic stress responses, and some unknown proteins with no ontology. Some were related to cell cycle and cell division, cell organization, regulation of transcription, transport, protein degradation, protein synthesis, protein targeting, large enzyme families, and development.

Plants have different defense mechanism in response to virus infection including the PAMP-triggered immunity pathway, the mitogen-activated protein kinase (MAPK) signaling pathway, and the ETI pathway. The transcriptomic data of full-length CTV and the severe phenotype indicated the activation of expression of genes related to ETI during plant–virus interactions, like TIR-NBS-LRR class, MAPK, and transcription factors (WRKY), suggesting the activation of these signaling pathways. The ETI response is usually followed by ROS production and could result in programmed cell death. Furthermore, the leucine-rich repeat (LRR) protein was found to be upregulated in these CTV isolates. These proteins have a role in hormone perception, pathogen response, and development (Jinn et al., 2000). HSPs that are essential elements in defense signaling and hypersensitive response (Kanzaki et al., 2003; Liu et al., 2004) were enriched in two CTV stem-pitting phenotypes. ROS are key signaling molecules that have been associated with abiotic and biotic stresses such as response to virus infection (Riedle-Bauer, 2000; Love et al., 2005). These molecules regulate programmed cell death (Levine et al., 1994); however, balance of these molecules is essential to regulate distinct plant defense layers. Plants have another defense mechanism involved in detoxification of ROS by production of enzymes including peroxidases, glutathione-S transferases, superoxide dismutases, and lipoxigenases. In our data, DEGs encoding these molecules and enzymes were detected.

Secondary metabolites in plants have roles in development, growth, and protection of plants from biotic and abiotic stresses (Zaynab et al., 2018). In full-length CTV, genes for secondary metabolites were overexpressed. These metabolites might have role in defense response against virus infection by cell wall thickening and programmed cell death mechanism, as Brlansky et al. (2002) showed thickened and collapsed cells in sections of an infected bark tissue of *Sweet orange* with a stem pitting isolate by light microscopy. However, for the severe CTV phenotype (CTVΔp33), up- and downregulated genes related to secondary metabolites were seen. Interestingly, most of the genes related to phenylpropanoids and lignin and lignans synthesis were downregulated in the severe phenotype. In a recent study, downregulation of some genes associated with the phenylpropanoid pathway such as cinnamoyl-CoA reductase (CCR), 4-coumarate: CoA ligase (4CL), caffeic acid O-methyltransferase (COMT), and phenylalanine ammonia-lyase (PAL) in the infected pitted stem with CTV-GFPΔp33, compared to the healthy stem, has been indicated (Sun and Folimonova, 2022). In full-length CTV, most of the DEGs related to defense mechanism and plant response were upregulated; however, in CTVΔp33, both up- and downregulated genes were detected. The overwhelmed and opposite expression pattern of plant response in CTVΔp33 suggests the occurrence of an extremely complex interaction between this CTV-variant and *C. macrophylla* that might result in production of severe symptoms. These findings suggest that the complexity of interactions between the CTV variants and *C. macrophylla* trees during viral infection might determine the intensity of symptoms and the extent of changes in expression profile. How complex is this interaction depends on the virus isolate and citrus variety, as it has been shown that the complexity of the host response to a virus infection is associated with the genetic variation in the host and in the virus (Love et al., 2005).

Auxin, cytokinin, and brassinosteroids have roles in xylem differentiation and phloem development (Dettmer et al., 2009; Wang, 2020). Brassinosteroid insensitive 2 (BIN2) and xyloglucan-xyloglucosyl transferase (TCH4) are involved in the brassinosteroid signaling pathway with a role in xylem differentiation (Nieminen et al., 2015). Early study demonstrated that the cytokinin signal transduction pathway regulates specification and cell proliferation during cambial activity (Dettmer et al., 2009; Wang, 2020). The signaling pathway maintains cell identities and inhibits protoxylem differentiation (Mähönen et al., 2006). Interestingly, our data indicated that genes related to the brassinosteroid signaling pathway and the cytokinin signal transduction pathway are differentially expressed in full-length CTV and Δp33.

The analysis of transcriptome data showed that xylem specification has been blocked by detecting several genes encoding xylem, cell wall and lignin degradation, and cell wall loosening enzymes. Furthermore, downregulation of transcription factors involved in regulation of xylem differentiation and genes involved in lignin biosynthesis provided additional evidence that the xylem differentiation and specification program has been shut off. It has been shown that MYB and NAC transcription factors are involved in regulation of xylem differentiation (Kubo et al., 2005; Zhong and Ye, 2012). Contrastingly, upregulation of genes encoding transcription factors associated with phloem and cambium development indicated the activation of this program in infected trees. KAN2, G2-like myb, NAM, DOF, MYB-related, Scarecrow, WUSCHEL-related homeobox, NAC domain-containing protein, bHLH, and the bZIP transcription factor family have been shown to be associated with phloem and cambium development (Zhang et al., 2011; Wang, 2020).

Our transcriptome data indicated DEGs related to CMT2, MET1, MBD6 and MBD7 in the moderate and severe stem-pitting phenotypes. The role of these genes in regulation of DNA methylation has been shown (Finnegan and Kovac, 2000), and the involvement of DNA methylation in early secondary vascular tissue (SVT) regeneration has been highlighted (Zhang et al., 2011). Furthermore, we detected induction of several DEGs encoding proteins associated with cell cycle re-entry such as chromatin remodeling factors, cyclin, and histone modification. It has been shown that the expression of enzymes involved in modification of histones changes during SVT regeneration (Zhang et al., 2011). Induction of genes encoding chromatin remodeling factors, cyclin, and histone modification are associated with cell cycle re-entry (Grafi et al., 2007; Zhang et al., 2011).

The microscopy analysis revealed the regeneration of new phloem in symptomatic stems. There was substantial phloem regeneration in the infected plants with the severe and moderate phenotypes that may compensate for the dysfunctional phloem. In comparison, there was a thin layer of phloem tissue in the mild phenotype and healthy plants, which means the lack of phloem regeneration in these plants.

**Figure 7:**
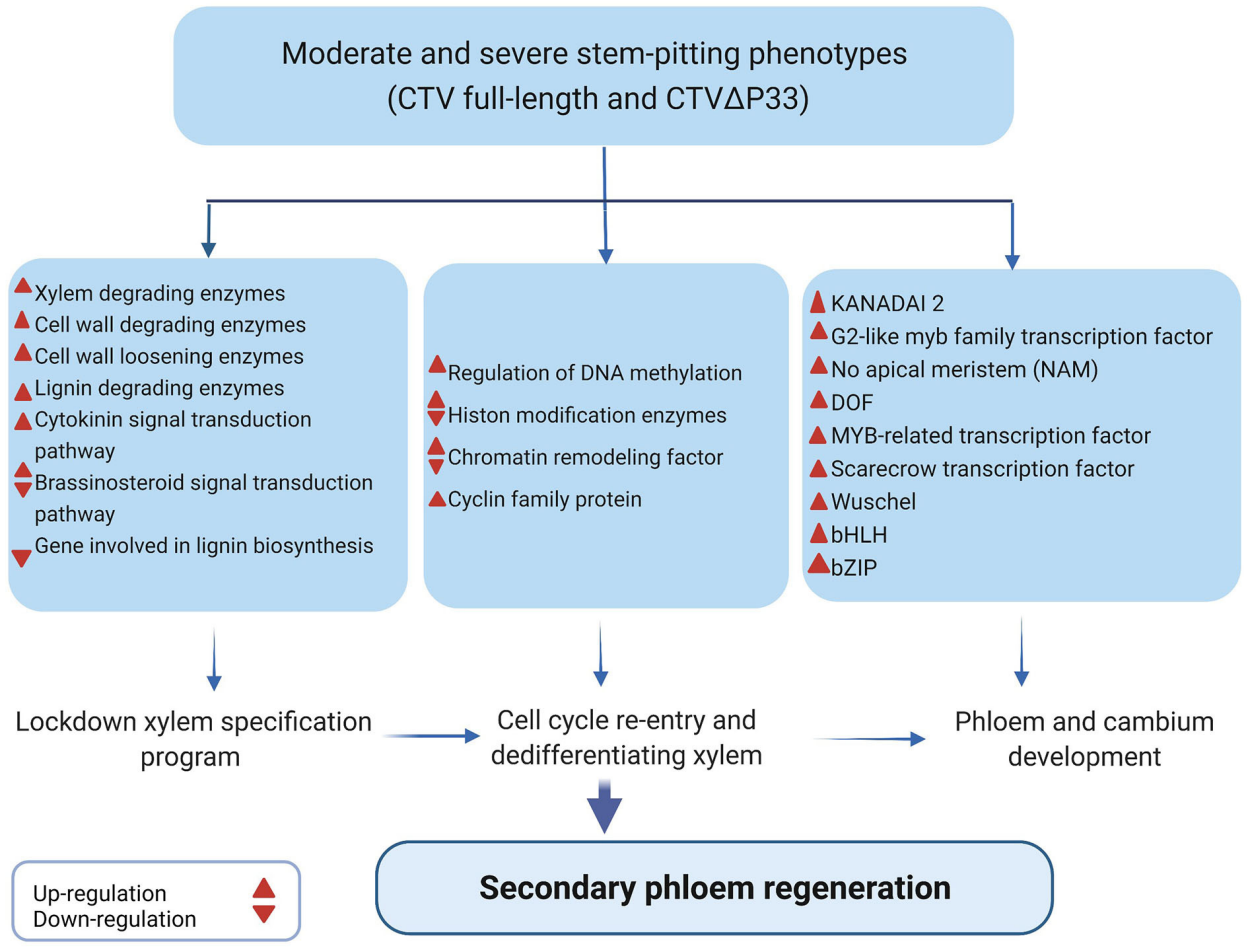
Illustration of gene expression changes and DEGs related to secondary phloem regeneration in infected plants with CTV moderate and severe stem-pitting phenotype during stem-pitting symptom development.

Based on the results, we suggest that CTV-plant interaction results in “developmental confusion,” and that during the stem pitting process, more phloem cells regenerate from maybe cambium cells that failed to differentiate to xylem cells or undifferentiated xylem cells because of infection with CTV stem pitting phenotypes (Figure 7). Phloem regeneration is a mechanism for plants to rebuild phloem cells after bark girdling in which immature xylem cells switch their fate and acquire their competence and dedifferentiate to produce phloem and cambium cells (Zhang et al., 2011). In this process, regulation of cellular plasticity is necessary for acquisition of competence of xylem cells (Birnbaum and Alvarado, 2008). It has been shown that cell cycle re-entry and chromatin remodeling followed by histone modification are necessary for regulation of cellular plasticity and maintaining this state (Zhang et al., 2011). Surprisingly, our data indicated enrichment of genes related to histone modification, cell cycle re-entry, and chromatin remodeling. Zhang et al. (2011) demonstrated that during SVT regeneration, genes related to xylem differentiation and specification are downregulated, and that phloem and cambium developmental programs are activated (Zhang et al., 2011). The transcriptome analysis data from this study indicated the opposite expression of hormones, enzymes, and transcription factors related to xylem specification and phloem development.

Finally, we conclude that there is a complex interaction between *C. macrophylla* and CTV specifically in the severe stem pitting phenotype. The overwhelmed but not efficient defense response of plants against infection in phloem tissues might result in dysfunctional phloem cells that interfere with the continuity of phloem network and result in disruption of its various transport roles. It has been shown that discontinuity in phloem cells like bark girdling or grafting leads to partial regeneration of missing tissues in the vasculature (Zhang et al., 2011). Here, we also suggest that in the dysfunctional area of *C. macrophylla* phloem cells because of CTV infection, plants start to fully or partially regenerate new phloem cells from undifferentiated xylem cells to compensate for non-functional cells. Dedifferentiated xylem cells that switched their fate and lost their cell wall (lignin and cellulose) are vulnerable cells resulting in enhanced viral infection. Once these dedifferentiated cells get infected with the virus, they do not develop into functional phloem sieve elements.

**Figure 8:**
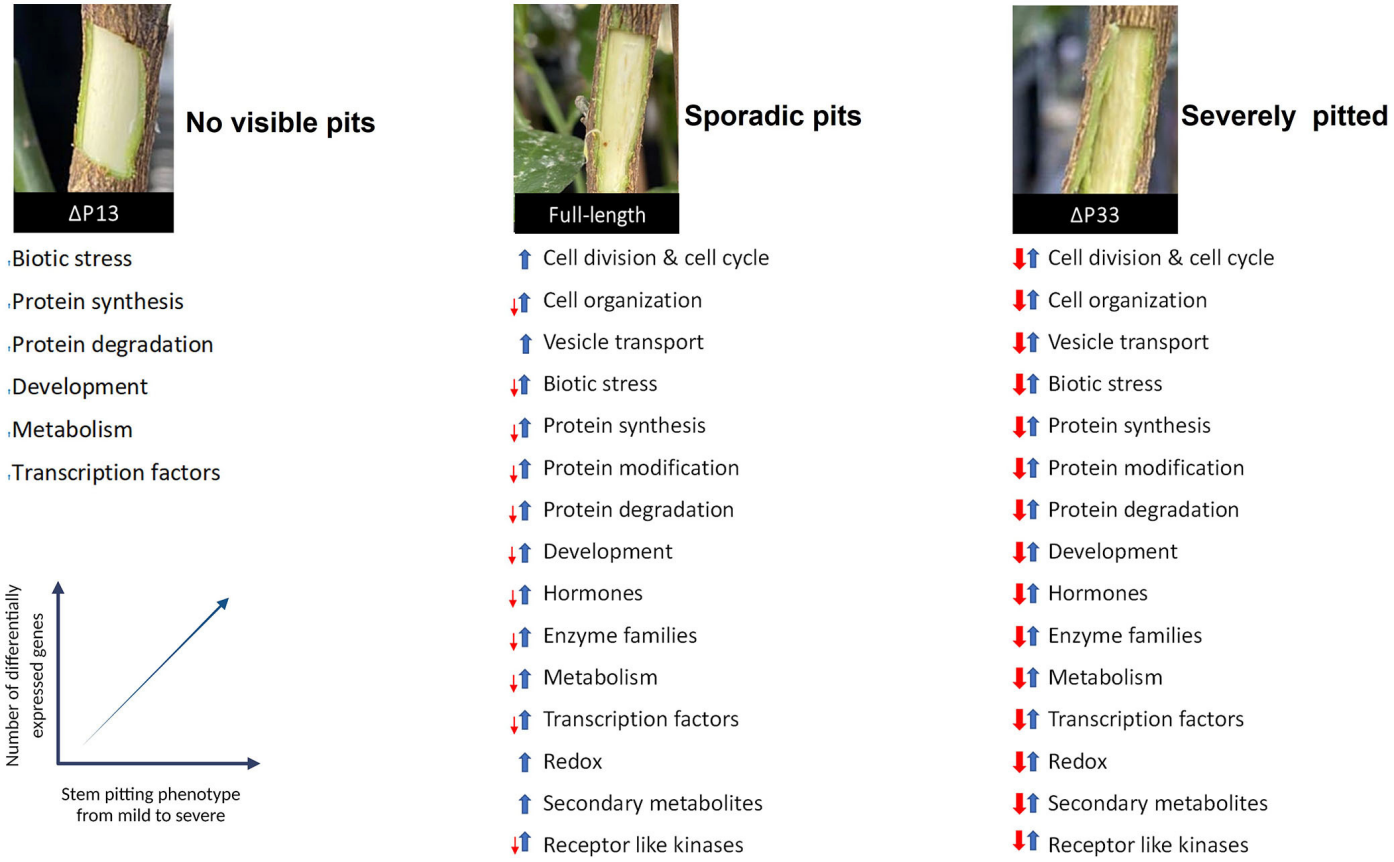
Transcriptome response of *Citrus macrophylla* trees infected with CTV wild-type and CTV different mutants which induce defined stem-pitting (SP) phenotypes from mild to severe. Degrees of SP symptom is correlated with the number of differentially expressed genes. Blue and red arrows represent the up and downregulation of genes related to each category, respectively. Thin arrows indicate that there are few numbers of DEGs in putative category.

Some questions will arise here regarding the role of the 13 and 33KDa proteins of CTV in stem pitting development. A previous study indicated that p33 is a CTV effector that modulates host immune response by triggering an effective defense response in infected cells and adjacent cells (Sun and Folimonova, 2019). Here, we demonstrated that the CTV mutant, in the absence of the p13 gene (CTVΔp13), tended to induce only a few numbers of DEGs related to general defense response and plant development and, consequently, induction of mild or non-visible stem pitting symptoms. In full-length CTV, which retained p13 and p33, plant response was moderate in terms of gene expression and symptom development. In contrast, extensive changes in gene expression and severe symptom production were observed in CTVΔp33 (with p13 retained) (Figure 8). These results suggest that both the p13 and p33 proteins have an important role in regulating stem pitting symptom development during plant-virus interaction by activating and modulating plant response, respectively. Therefore, in infected plants with full-length CTV, which shows moderate symptoms and less DEGs compared to the severe phenotype, the expression of the p13 protein activates plant response and induces DEGs, but the plant response is modulated and not overwhelmed because of the expression of the p33 protein in this isolate.

Collectively, this study provided a detailed overview of transcriptomes of defined stem pitting phenotypes and evidence of secondary phloem regeneration during stem pitting development, suggesting that phloem regeneration is a key element that contributes to the development of stem pitting symptoms. The results of this study help us to get further insights into the molecular mechanisms underlying the stem pitting phenomenon and enhance our knowledge of the complex interaction between viruses and plants during symptom development.

## Data availability statement

The RNA-seq raw data of all samples are available at NCBI BioProject PRJNA851012 with accession number of 4 objects (SRR19781992, SRR19781993, SRR19781994, and SRR19781995).

## Author contributions

AL and MK conceived and designed the study. AL supervised the study, aided with the interpretation of the results and helpful discussions, and revised the manuscript. MK performed the experiment, analyzed the data, and wrote the manuscript. KW processed the RNA-seq data, helped to conduct the data analysis, and write some parts of the methods of the manuscript. MD and CE-M helped to revise the manuscript. MD provided support for data processing. All authors contributed to the article and approved the final version of the manuscript.

## Funding

This study was supported by the Florida State Legislative Funding for the UF/IFAS Citrus Initiative. MK was supported by Hunt Brothers Graduate Student Assistantship.

## Conflict of interest

The authors declare that the research was conducted in the absence of any commercial or financial relationships that could be construed as a potential conflict of interest.

## Publisher's note

All claims expressed in this article are solely those of the authors and do not necessarily represent those of their affiliated organizations, or those of the publisher, the editors and the reviewers. Any product that may be evaluated in this article, or claim that may be made by its manufacturer, is not guaranteed or endorsed by the publisher.

## Supplementary material

Supplementary Figure 1 Graph shows the CT value for *Citrus macrophylla* trees infected with CTVΔp13, full-length CTV, and CTVΔp33 at 8 months post-inoculation.

Supplementary Figure 2 MapMan visualization of differentially expressed genes related to biotic stress, cell function, and cellular response overview in CTVΔp13 vs. healthy. Blue and red squares indicate up-and downregulation of genes, respectively.

Supplementary Figure 3 MapMan visualization of differential expressed genes related to cell function in full-length CTV vs Healthy and CTVΔp33 vs Healthy. Blue and Red squares indicate up-and down regulation of genes, respectively.

Supplementary Figure 4 MapMan visualization of differential expressed genes related to cellular response in full-length CTV vs Healthy and CTVΔp33 vs Healthy. Blue and Red squares indicate up-and down regulation of genes, respectively.

Supplementary Figure 5 MapMan visualization of differential expressed genes related to regulation in full-length CTV vs Healthy and CTVΔp33 vs Healthy. Blue and Red squares indicate up-and down regulation of genes, respectively.

Supplementary Figure 6 MapMan visualization of differential expressed genes related to metabolism in full-length CTV vs Healthy and CTVΔp33 vs Healthy. Blue and Red squares indicate up-and down regulation of genes, respectively.

Supplementary Figure 7 MapMan visualization of differential expressed genes related to large enzyme families in full-length CTV vs Healthy and CTVΔp33 vs Healthy. Blue and Red squares indicate up-and down regulation of genes, respectively.

Supplementary Figure 8 MapMan visualization of differential expressed genes related to transcription factors in full-length CTV vs Healthy and CTVΔp33 vs Healthy. Blue and Red squares indicate up-and down regulation of genes, respectively.

Supplementary Figure 9 MapMan visualization of differential expressed genes related to proteasome and autophagy in full-length CTV vs Healthy and CTVΔp33 vs Healthy. Blue and Red squares indicate up-and down regulation of genes, respectively.

Supplementary Figure 10 MapMan visualization of differential expressed genes related to protein targeting in full-length CTV vs Healthy and CTVΔp33 vs Healthy. Blue and Red squares indicate up-and down regulation of genes, respectively.

Supplementary Figure 11 MapMan visualization of differential expressed genes related to receptor-like kinases in full-length CTV vs Healthy and CTVΔp33 vs Healthy. Blue and Red squares indicate up-and down regulation of genes, respectively.

Supplementary Table 1 Differentially expressed genes (DEGs) encoding proteins involved in plant-pathogen interaction, leucine-rich repeats receptor-like kinases, secondary metabolites, and phytohormones, and genes involved in secondary phloem regeneration for *Citrus macrophylla* trees infected with full-length CTV and CTVΔp33 (moderate and severe stem-pitting phenotypes).
