# Supplementary figures and images for: Comparative transcriptome analysis of Citrus macrophylla tree infected with Citrus tristeza virus stem pitting mutants provides new insight into the role of phloem regeneration in stem pitting disease
Source: Front Plant Sci. 2022 Oct 4;13:987831. doi: 10.3389/fpls.2022.987831 (PMC9577373; doi:10.3389/fpls.2022.987831)

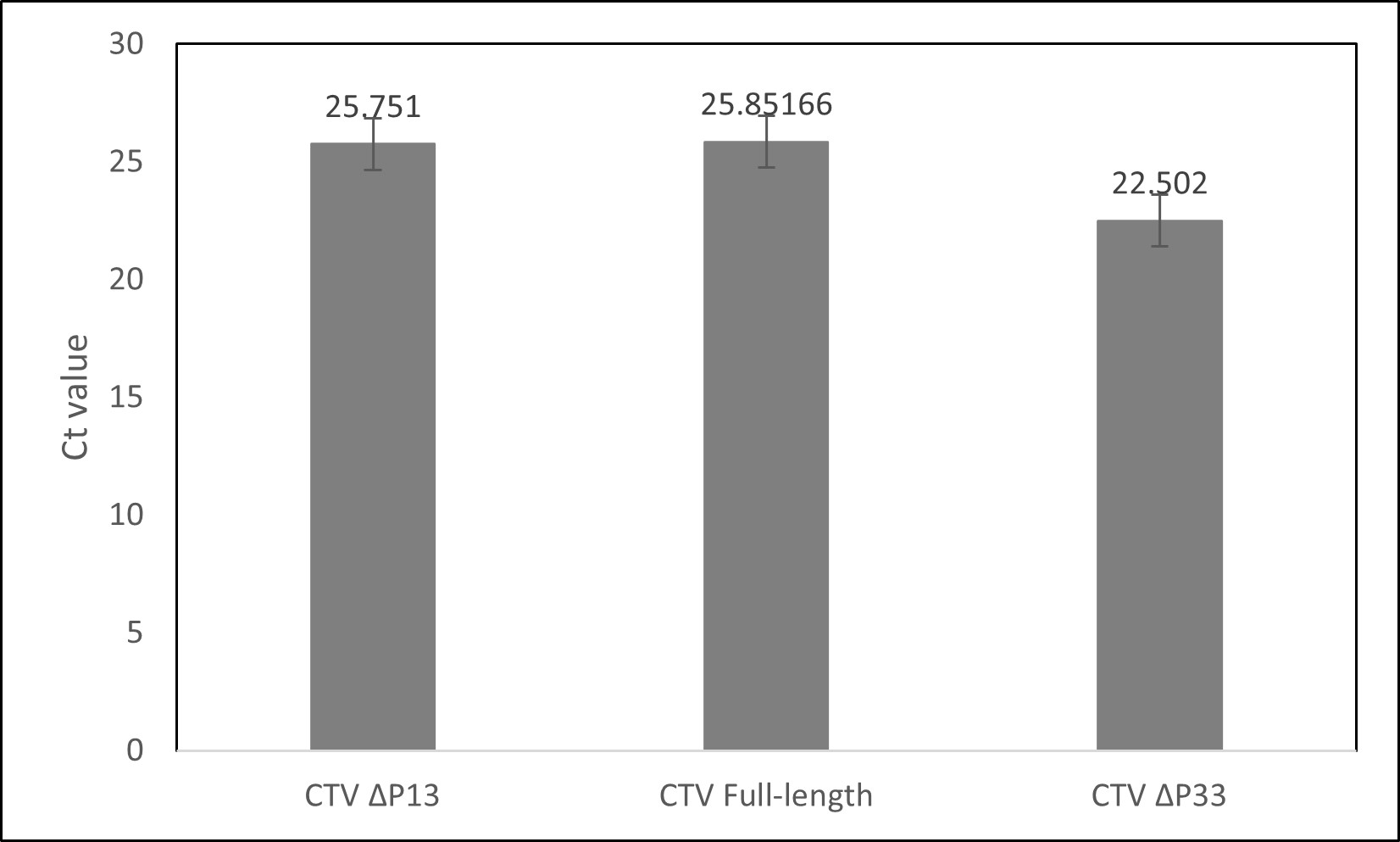

Supplement: Supplementary Figure 1 — Graph shows the CT value for Citrus macrophylla trees infected with CTVΔp13, full-length CTV, and CTVΔp33 at 8 months post-inoculation. [file Image_1.JPEG]

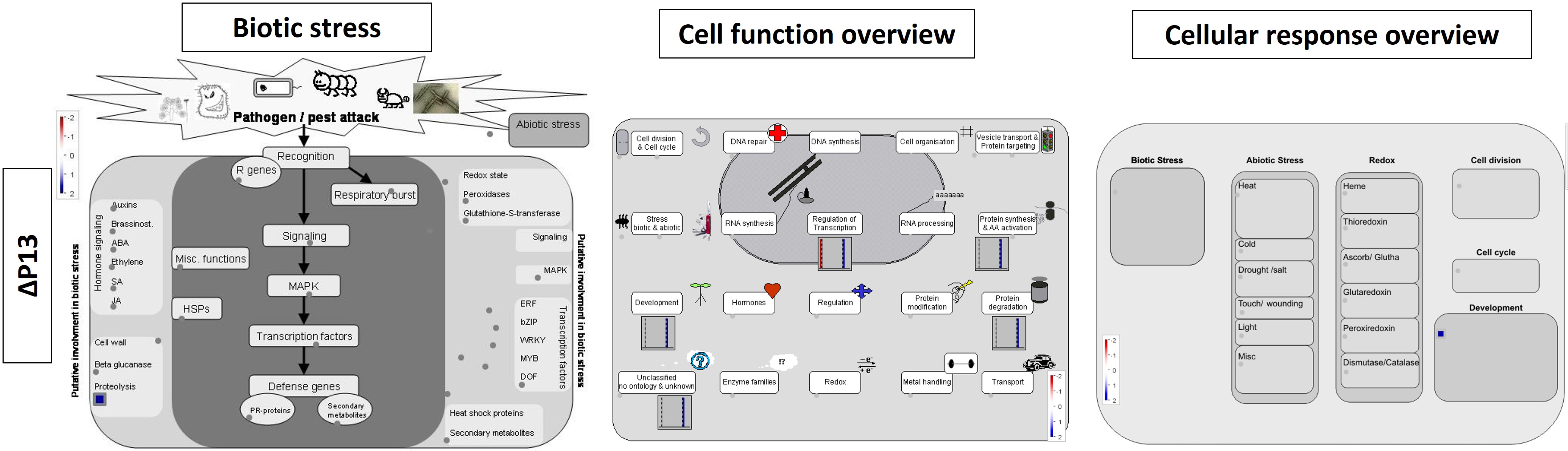

Supplement: Supplementary Figure 2 — MapMan visualization of differentially expressed genes related to biotic stress, cell function, and cellular response overview in CTVΔp13 vs. healthy. Blue and red squares indicate up-and downregulation of genes, respectively. [file Image_2.JPEG]

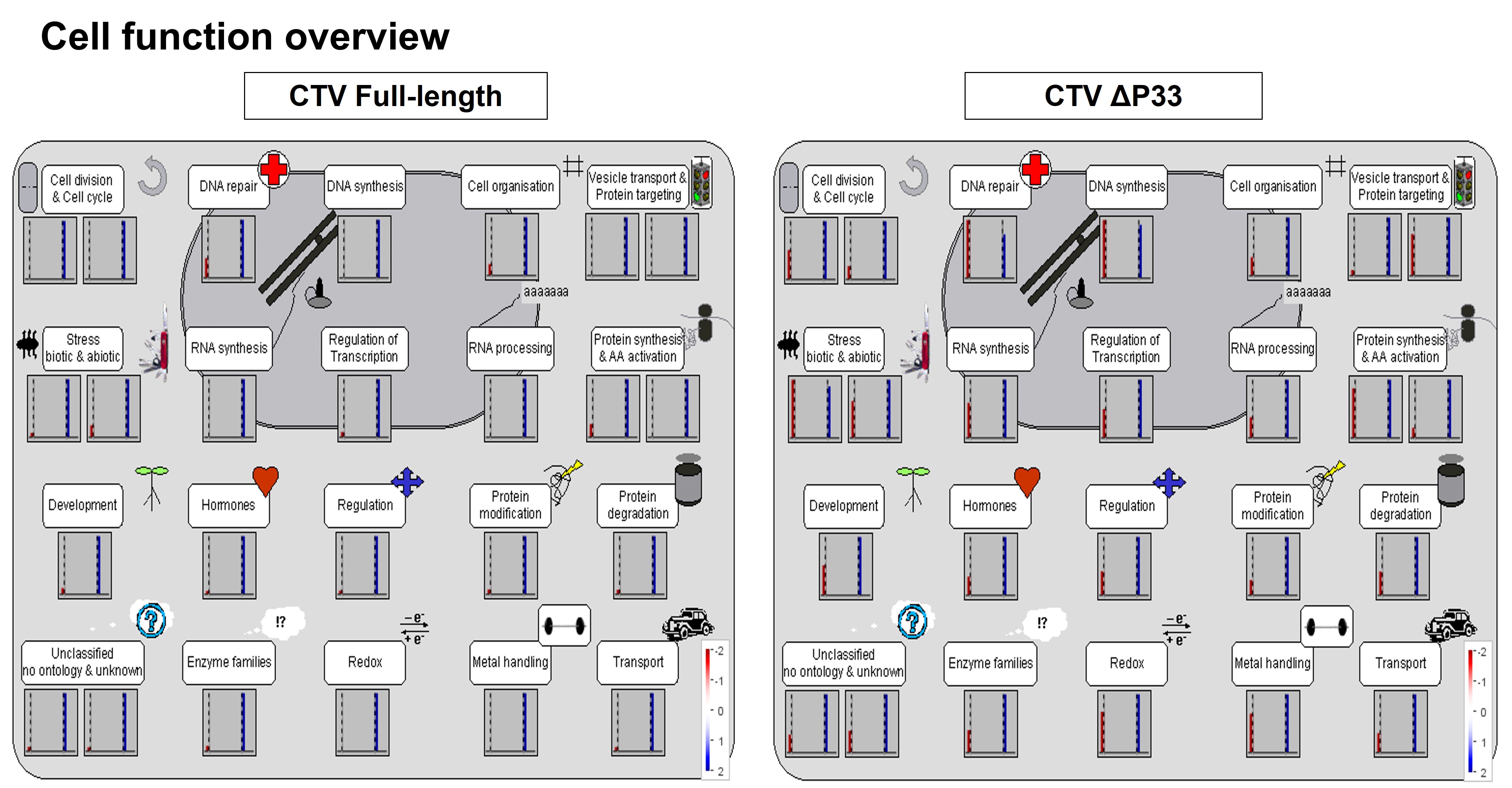

Supplement: Supplementary Figure 3 — MapMan visualization of differential expressed genes related to cell function in full-length CTV vs Healthy and CTVΔp33 vs Healthy. Blue and Red squares indicate up-and down regulation of genes, respectively. [file Image_3.JPEG]

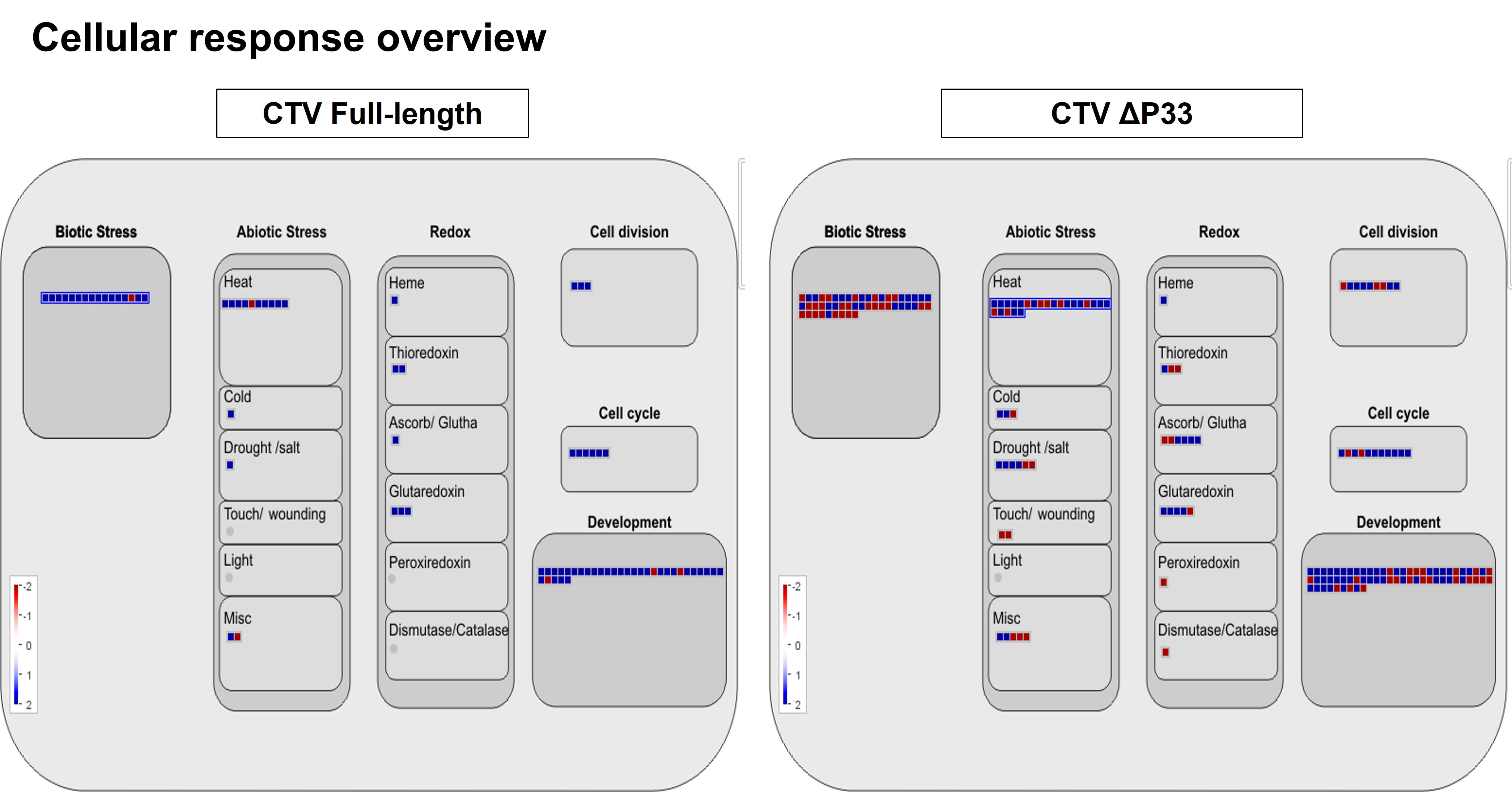

Supplement: Supplementary Figure 4 — MapMan visualization of differential expressed genes related to cellular response in full-length CTV vs Healthy and CTVΔp33 vs Healthy. Blue and Red squares indicate up-and down regulation of genes, respectively. [file Image_4.JPEG]

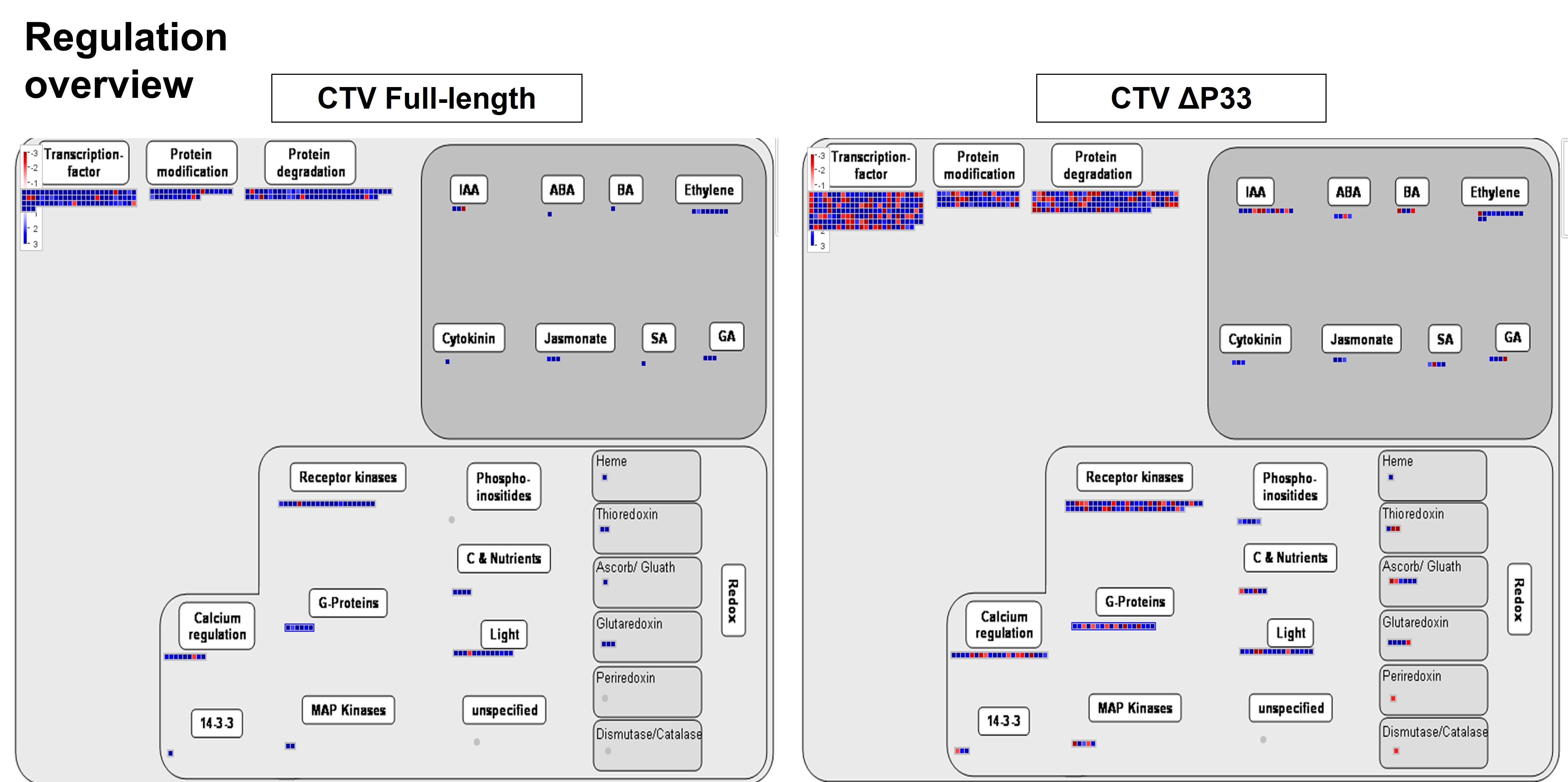

Supplement: Supplementary Figure 5 — MapMan visualization of differential expressed genes related to regulation in full-length CTV vs Healthy and CTVΔp33 vs Healthy. Blue and Red squares indicate up-and down regulation of genes, respectively. [file Image_5.JPEG]

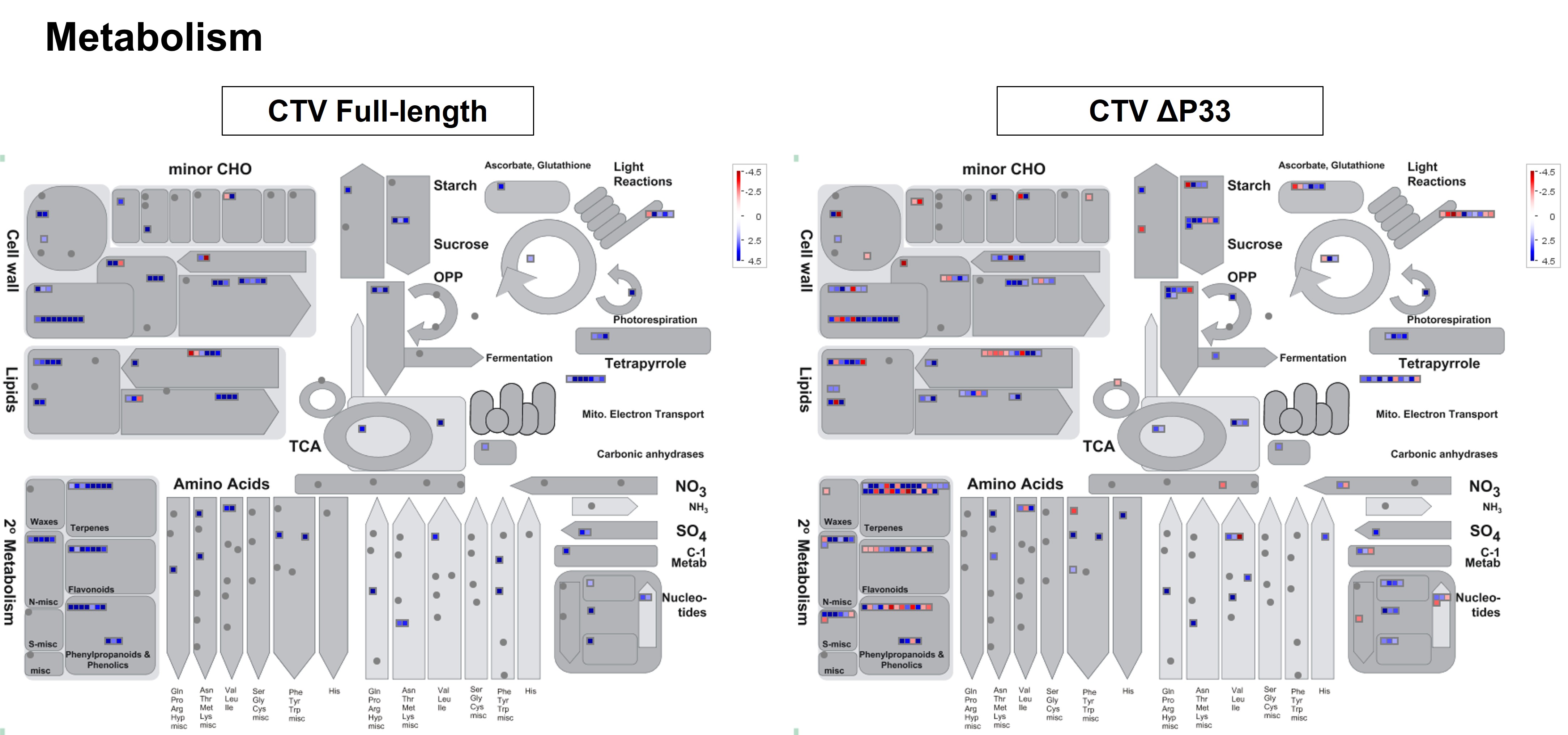

Supplement: Supplementary Figure 6 — MapMan visualization of differential expressed genes related to metabolism in full-length CTV vs Healthy and CTVΔp33 vs Healthy. Blue and Red squares indicate up-and down regulation of genes, respectively. [file Image_6.JPEG]

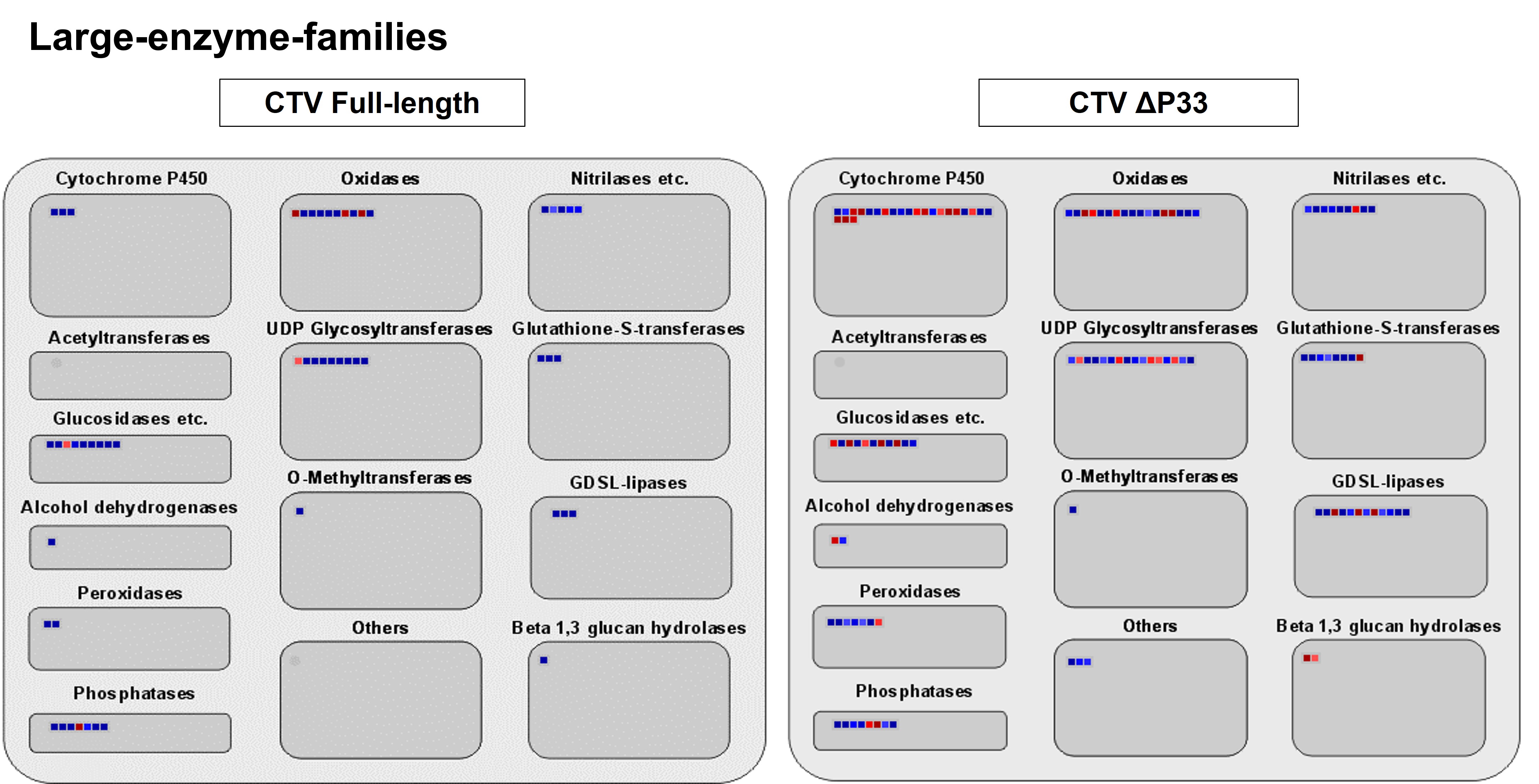

Supplement: Supplementary Figure 7 — MapMan visualization of differential expressed genes related to large enzyme families in full-length CTV vs Healthy and CTVΔp33 vs Healthy. Blue and Red squares indicate up-and down regulation of genes, respectively. [file Image_7.JPEG]

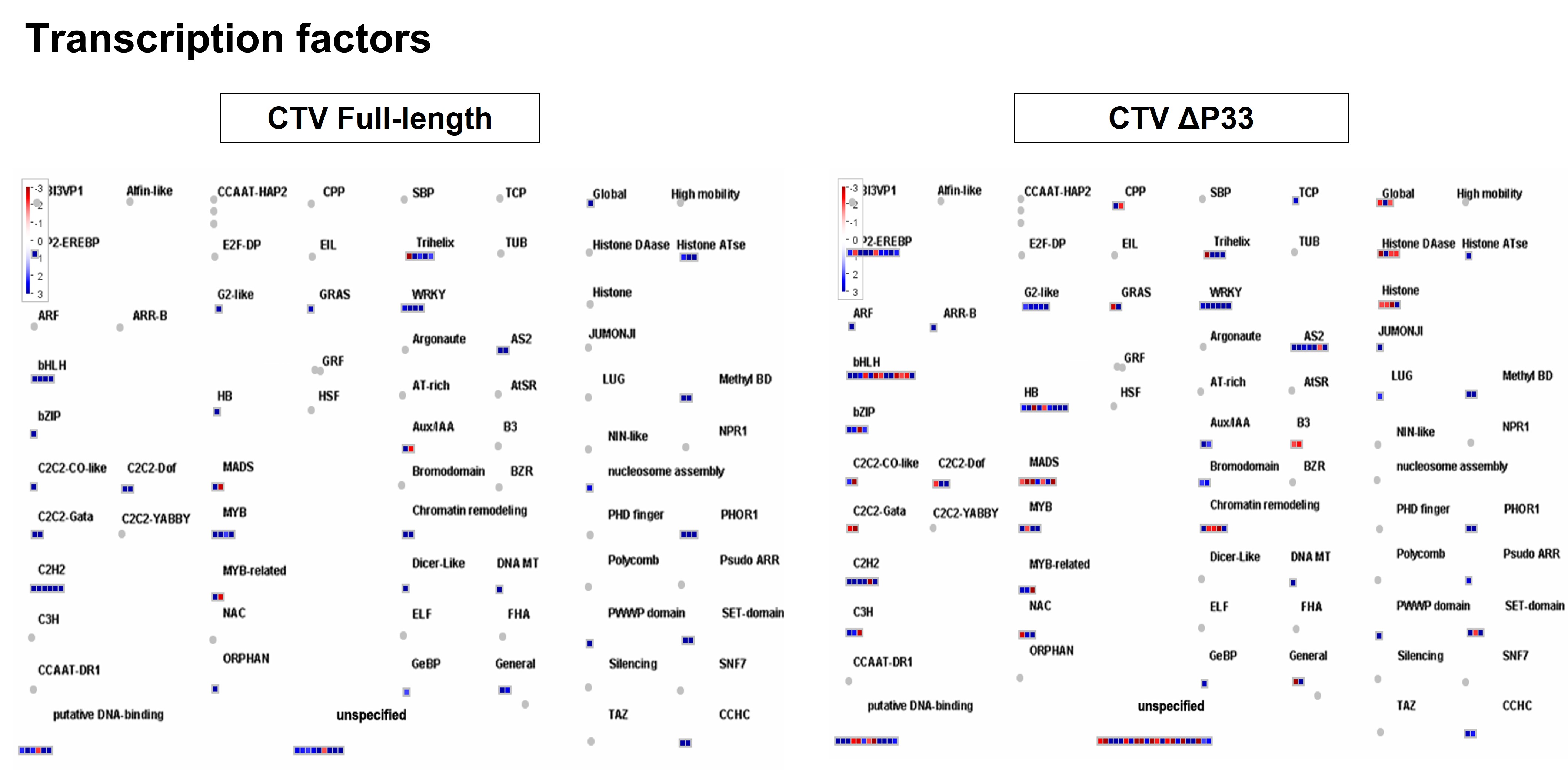

Supplement: Supplementary Figure 8 — MapMan visualization of differential expressed genes related to transcription factors in full-length CTV vs Healthy and CTVΔp33 vs Healthy. Blue and Red squares indicate up-and down regulation of genes, respectively. [file Image_8.JPEG]

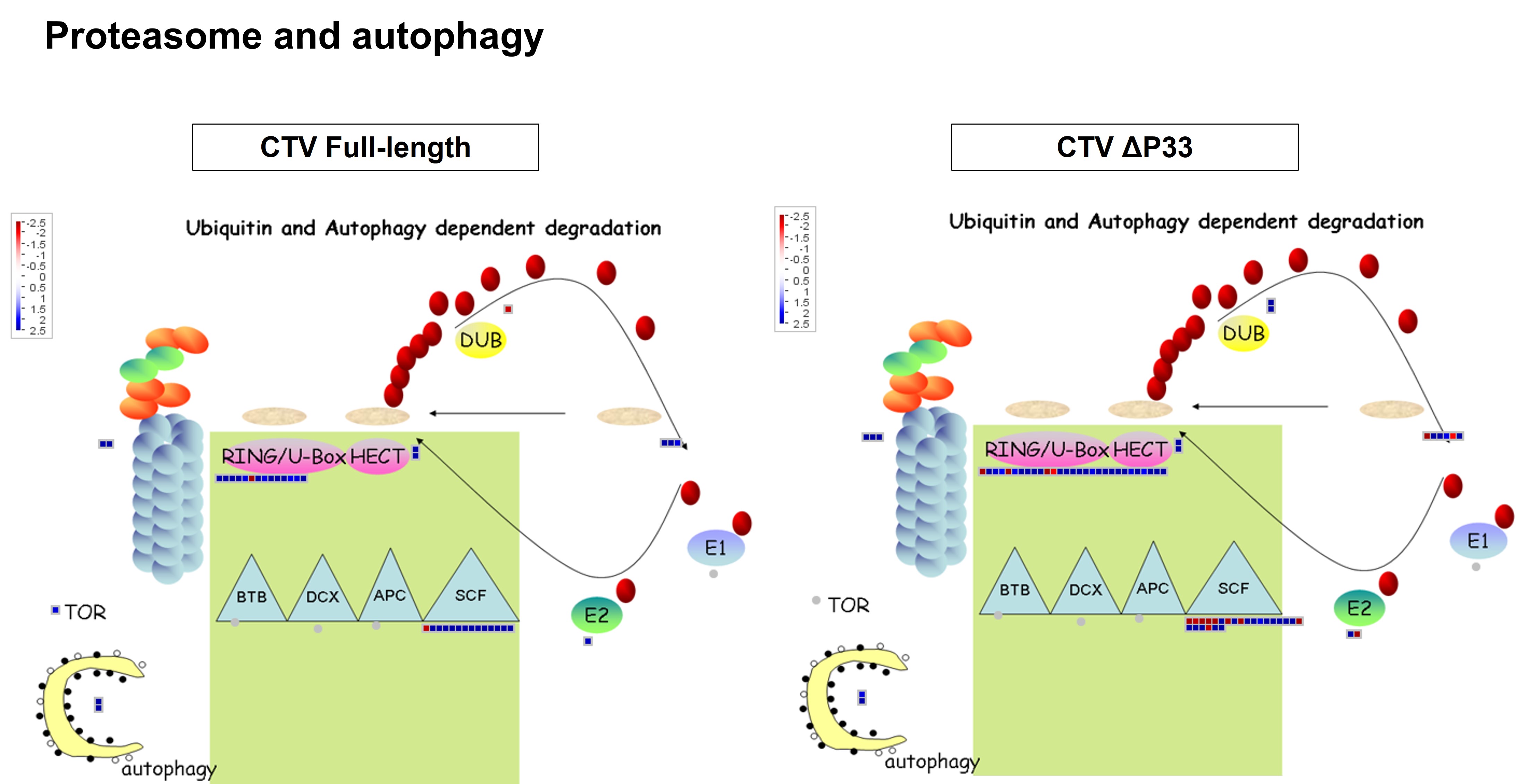

Supplement: Supplementary Figure 9 — MapMan visualization of differential expressed genes related to proteasome and autophagy in full-length CTV vs Healthy and CTVΔp33 vs Healthy. Blue and Red squares indicate up-and down regulation of genes, respectively. [file Image_9.JPEG]

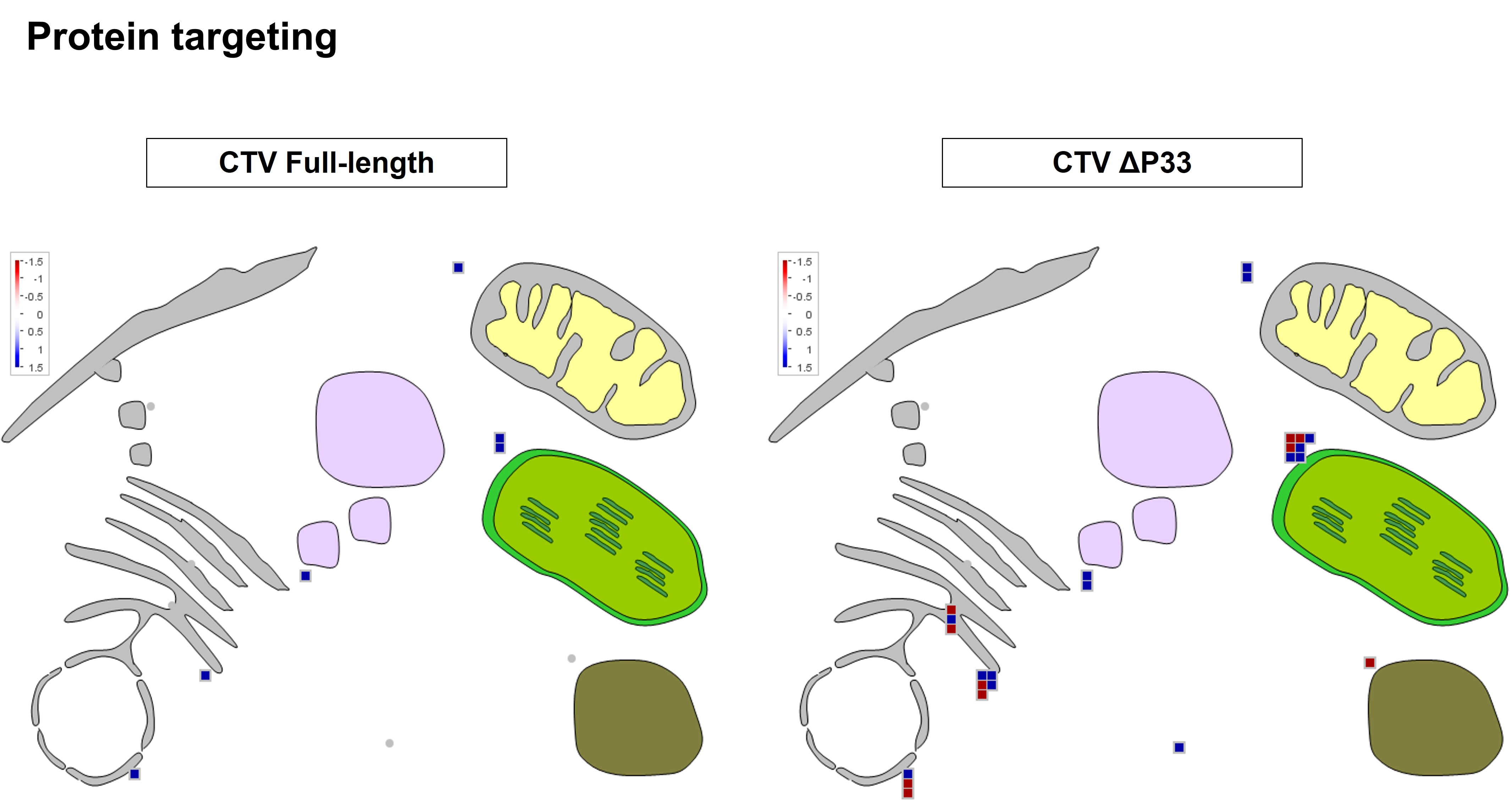

Supplement: Supplementary Figure 10 — MapMan visualization of differential expressed genes related to protein targeting in full-length CTV vs Healthy and CTVΔp33 vs Healthy. Blue and Red squares indicate up-and down regulation of genes, respectively. [file Image_10.JPEG]

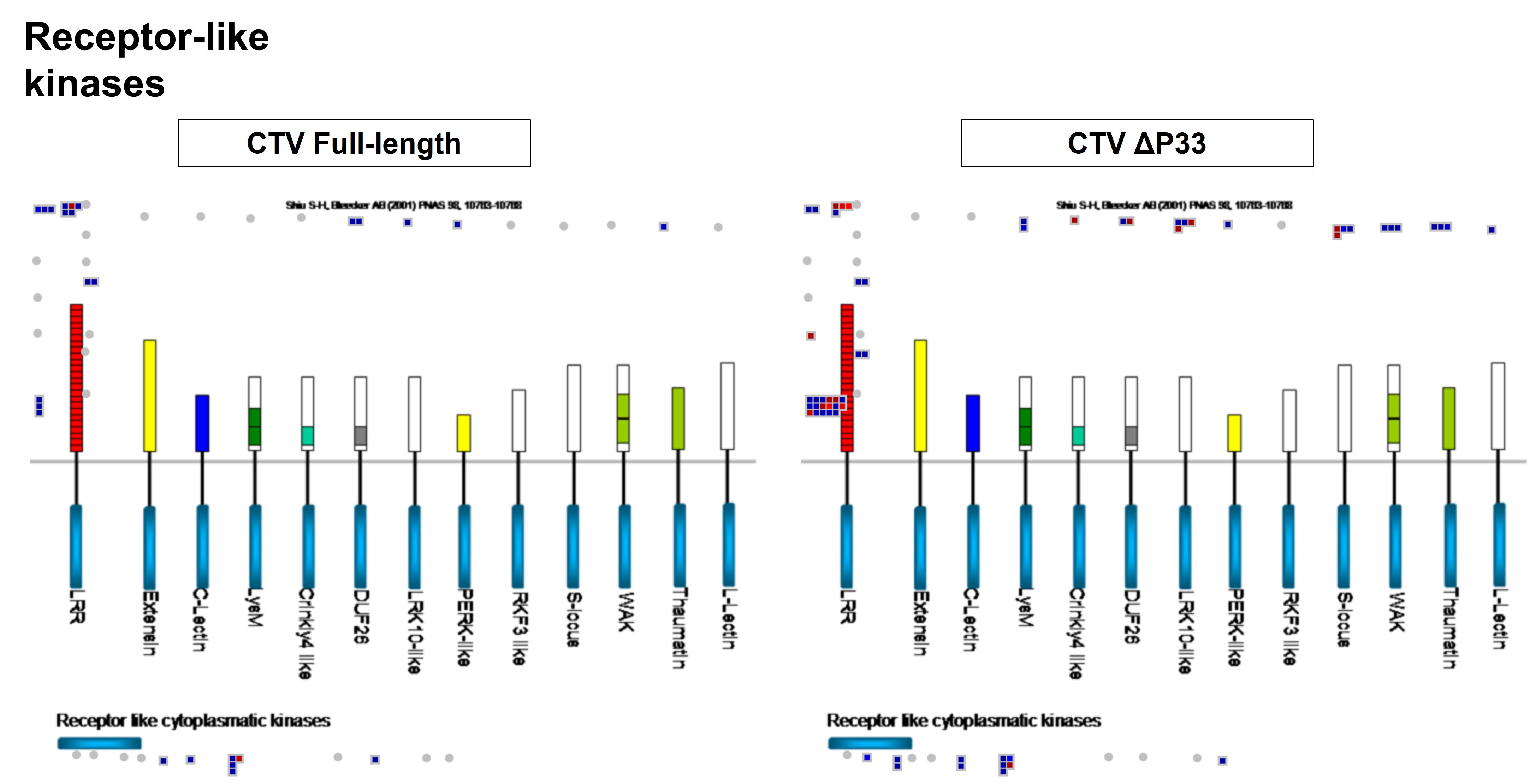

Supplement: Supplementary Figure 11 — MapMan visualization of differential expressed genes related to receptor-like kinases in full-length CTV vs Healthy and CTVΔp33 vs Healthy. Blue and Red squares indicate up-and down regulation of genes, respectively. [file Image_11.JPEG]
